# Genomic characterization of bacteriophage BI-EHEC infecting strains of Enterohemorrhagic *Escherichia coli*

**DOI:** 10.1186/s13104-021-05881-5

**Published:** 2021-12-20

**Authors:** Marta Nisita Dewanggana, Diana Elizabeth Waturangi, Yogiara

**Affiliations:** Faculty of Biotechnology, Atma Jaya Catholic University of Indonesia, Jalan Jenderal Sudirman 51, Jakarta, 12930 Indonesia

**Keywords:** Food preservatives, Foodborne, Biocontrol agent, Bacteriophage, Genome sequence

## Abstract

**Objective:**

The aims of this research were to determine the genomic properties of BI-EHEC to control Enterohemorrhagic *Escherichia coli* (EHEC), which was isolated from previous study. Genomic analysis of this phage is essential for the assessment of this bacteriophage for further application as food preservatives.

**Results:**

Genome of BI-EHEC was successfully annotated using multiPhATE2. Structural and lytic cycle-related proteins such as head, tail, capsid, and lysozyme (lysin) were annotated. The phylogenetic tree of tail fiber protein and BRIG results showed that BI-EHEC was similar to phages of the same host in the bacteriophage genome database. There were no indications of virulence properties, antibiotic resistance genes and lysogenic protein among annotated genes which implied BI-EHEC followed a lytic life cycle. PHACTS analysis was done to confirm this notion further and yielded a lytic cycle result. Further analysis using CARD found that BI-EHEC does not contain residual ARGs per recommended parameter. Furthermore, BI-EHEC confirmed as lytic bacteriophage, making it a good candidate for biocontrol agent.

**Supplementary Information:**

The online version contains supplementary material available at 10.1186/s13104-021-05881-5.

## Introduction

Foodborne disease is often caused by consuming food contaminated by bacteria, one of the foodborne bacteria is EHEC. Conventional preservation methods has many disadvantages, such as the loss of nutritional and organoleptic value [1, 2]. Bacteriophage can be used as an alternative approach. Bacteriophage has two different life cycles. Lytic life cycle enables bacteriophages to lyse the bacterial host and create progeny, it is preferable to be used as biocontrol agent to minimize the probability for horizontal gene transfer. While lysogenic life cycle only enables DNA replication in the host. Lytic bacteriophage is [3].

Our previous study isolated bacteriophage from bovine intestine referred as BI-EHEC, found to be effective in controlling EHEC with 91.02% of reduction [4]. However, genomic properties analysis of BI-EHEC has not been done. In this research, we would like to use in silico approach to determine that BI-EHEC possessed certain criteria as a promising candidate for biocontrol agent.

## Main text

### Methods

#### Bacteriophage enrichment and purification

EHEC were growth in Luria Bertani (LB) agar media (OXOID), incubated at 37 °C overnight, then stored in a refrigerator at 4 °C. Host bacteria were growth into LB broth media incubated in water bath shaker (Lab Companion) at 120 rpm, 37 °C overnight. BI-EHEC from stock solution was enriched by adding 1.63 ± 0.65 × 10^10^ PFU/mL of phage solution and 10^8^ CFU/mL (OD600 = 0.132) of its host bacteria into a fresh LB broth media (OXOID). The suspension was then incubated using a water bath shaker at 120 rpm, 37 °C, overnight, then the suspension was centrifuged (Eppendorf) at 5488×*g* for 15 min. The pellet was removed, and the supernatant was taken to be filtered with a 0.22 μm microfilter (Himedia, Mumbai, India). The purified bacteriophage stock can be kept at 4 °C with the addition of Ringer Solution (OXOID) (1:9 v/v) for further steps. Additionally, agar overlay method was performed to verify the activity and presence of BI-EHEC by observing a clear plaque [5–7].

#### Isolation of bacteriophage genomic material

As much as 5 μL of DNase I (Geneaid) were added to 1.63 ± 0.65 × 10^10^ PFU/mL of purified bacteriophage, then incubated at 37 °C for 30 min. Then 6 μL of EDTA 0.05 M, 10 μL of 1% sodium dodecyl sulfate (SDS) and 6 μL of proteinase K (Geneaid) (10 mg/mL) were added. The mixture was incubated at 37 °C for 1 h. Then 600 μL of phenol–chloroform-isoamyl alcohol solution (25:24:1) was added and centrifuged (Thermo) at 2655*g* for 5 min. The upper phase was taken into a new microtube, mixed with 500 μL of chloroform-isoamyl alcohol solution (24:1), and centrifuged at 2655×*g* for 5 min. The upper phase was taken into a new microtube. A 3M sodium acetate pH 5.2 (1:10) solution followed by isopropyl alcohol (1:1) (MERCK) was added to the mixture and incubated in ice bath for 15 min. Then the suspension was centrifuged at 17,949×*g* for 10 min, and the supernatant was removed. About 700 μL of 70% of ethanol was added to the pellet, and the mixture was centrifuged again at 17,949×*g* for 10 min. The supernatant was removed, and the pellet was dried. A 50 μL of nuclease-free water (NFW) (Qiagen) solution was added to the pellet for DNA storage at 4 °C [8].

#### Next-generation sequencing (NGS)

gDNA sequences obtained from bacteriophage genomic isolation were sent to PT Genetika Science Indonesia for NGS using Oxford Nanopore Technologies (MinKNOW 20.06.9). Base Calling was done using Guppy 4.0.11 high accurate mode. Raw NGS data were filtered using Filtlong v.0.2.0, utilizing the default parameter without an external reference [9]. *De novo* assembly was done with Flye v.2.8.3 using the default parameter for Oxford nanopore input [10] on the resulting Filtlong fasta. Medaka 1.2.0 (default parameter) [11] was used to polish the assembled genome. The resulting fasta was treated as the complete genome assembly for BI-EHEC.

#### Bioinformatic analysis

Genome annotations were carried out with multiPhATE2, using default databases (Phantome, pVOGs) and supporting databases (NCBI virus genomes, NCBI Swissprot, CAZy) [12]. A phylogenetic tree of tail fiber protein was constructed using MEGAX (nucleotide sequence) [13, 14]. BLAST analysis was carried out to determine the similarity BI-EHEC most resembles [15]. Two additional bacteriophages were chosen from NCBI database to be compared with BI-EHEC using BRIG [16]. Virulence (*eae, lpf, stx*) and lysogenic (*int*, *xis*) genes were also compared with BI-EHEC via BRIG. Further analysis was done using CARD [17] to study the possible presence of antimicrobial resistance genes (ARGs). PHACTS [18] was performed to determine the life cycle of BI-EHEC.

### Results

#### Bacteriophage annotation

The BI-EHEC (GenBank accession number OL505078) is composed of 151.425 bp with 39% GC content. It has 12 encoded tRNA regions and 352 open reading frames (ORF). Genes associated with cell lysis, assembly, and packaging during the end of the lytic cycle were annotated. It includes putative T4-like lysozyme (EC 3.2.1.17), tail fiber assembly protein, gpH, and terminases. Other results include parts associated with bacteriophage structures [3, 19]. Complete annotation can be seen in Additional file 1: Table S1, genome map in Additional file 2: Figure S1 and selected results in Table 1.

**Table 1:** Notable BI-EHEC annotation results

CDs	Annotation	Source of organism	Putative role
BI-EHEC-60	Major capsid protein	*Bacillus* phage VMY22	Structural
BI-EHEC-89	Putative bacteriophage T4-like lysozyme	*Escherichia* phage phAPEC8	Cell lysis
BI-EHEC-154	Phage tail sheath protein, baseplate wedge subunit	Uncultured Mediterranean phage uvMED-GF-U-MedDCM-OCT-S28-C30, *Edwardsiella* phage PEi26	Structural
BI-EHEC-157	Putative terminase large subunit	*Escherichia* phage phAPEC8	Packaging
BI-EHEC-162	Putative head stabilization/decoration protein	*Escherichia* phage phAPEC8	Structural
BI-EHEC-170	Putative tail tube	*Enterobacteria* phage ECGD1	Structural
BI-EHEC-186	Putative gpH domain protein	*Escherichia* phage phAPEC8	Assembly
BI-EHEC-187	Putative tail fiber assembly protein	*Escherichia* phage phAPEC8	Assembly
BI-EHEC-190	Putative phage tail fiber protein	*Escherichia* phage phAPEC8	Structural
BI-EHEC-251	Terminase, gp5, gp74	*Listeria* phage LMTA-57, *Listeria* virus P100	Packaging
BI-EHEC-319	Terminase small subunit	*Geobacillus* phage GBSV1	Packaging

Resulting annotations from multiPhATE2 that associated with cell lysis, assemblies, packaging and phage structures

#### Phylogenetic analysis of tail fiber protein

**Fig. 1:**
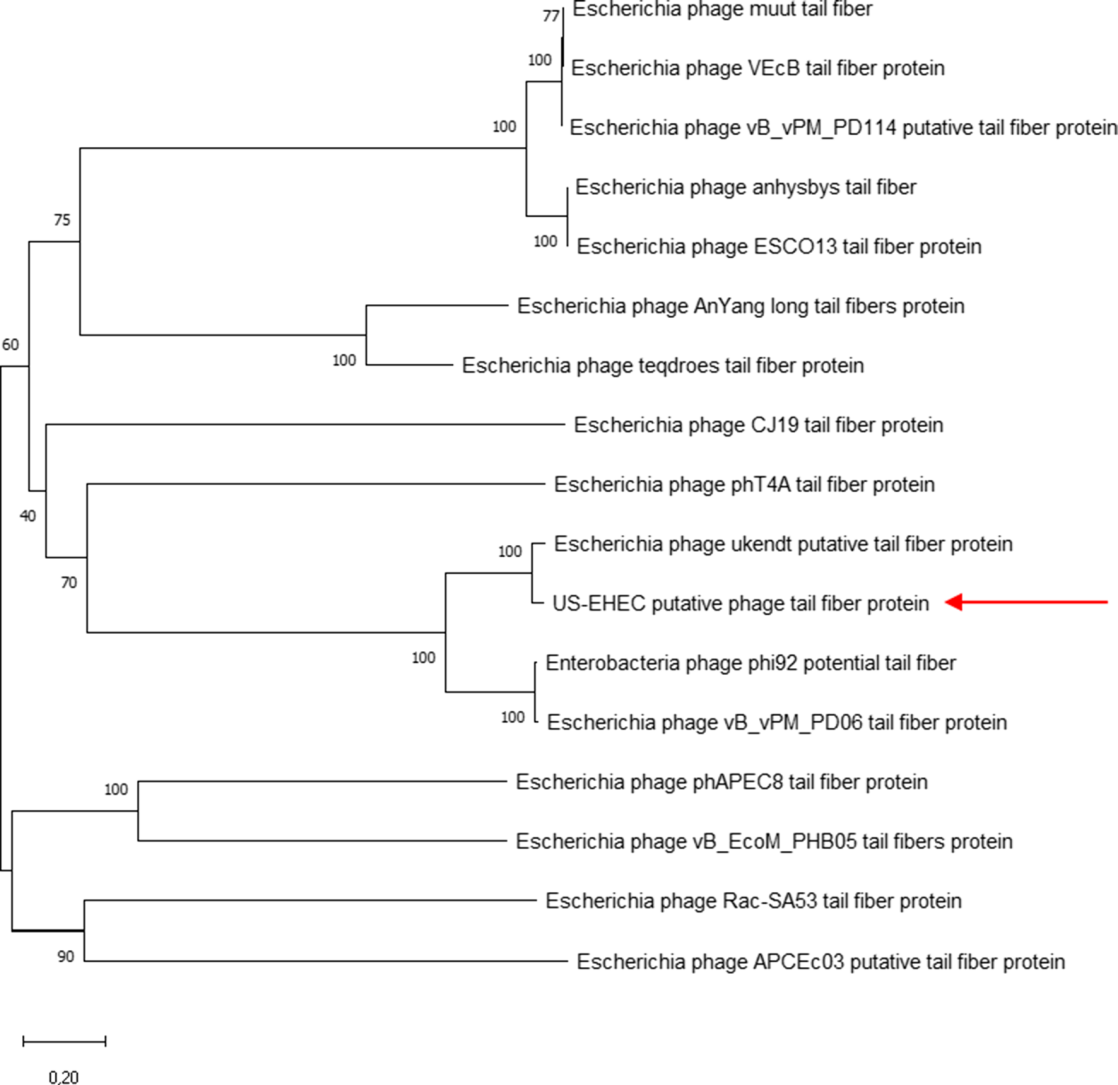
Unrooted phylogenetic tree of BI-EHEC tail fiber protein and other related phages. Phylogenetic tree was based on 100 replications on bootstrap percentage analysis. Homology between species in all tail fiber protein sequences used was indicated by the bar below the figure (20% homology). BI-EHEC used in this analysis was indicated by the arrow

**Fig. 2:**
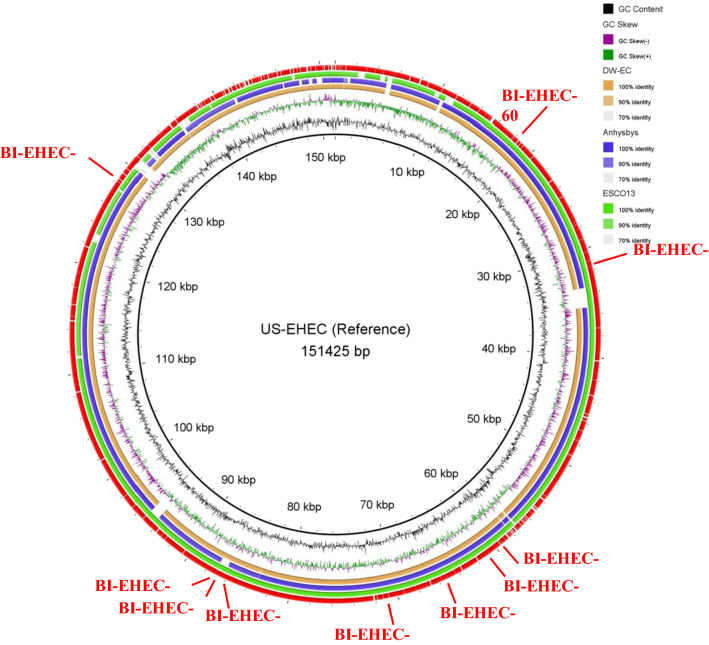
Comparative genomic analysis of BI-EHEC and the other bacteriophages. The inner circle is the BI-EHEC genome as a reference. The dataset used for BRIG analysis using BI-EHEC as reference were: Unpublished bacteriophage DW-EC of ETEC (orange), *E. coli* phage anhysbys (blue) and *E. coli* phage ESCO13 (green). Additional annotation ring (red) was added, and the contents can be viewed on Table 1. Colored rings represent regions that were present on both reference and compared genomes. Conversely, white gaps indicate that certain sections were not present on BI-EHEC

Sixteen tail fiber protein sequences were obtained from NCBI databases to be compared with BI-EHEC [see Additional file 3: Table S2]. BI-EHEC tail fiber protein showed high similarity with *Escherichia* phage ukendt tail fiber protein (Figs. 1, 2).

#### BLAST and BRIG

BLAST (BLASTn) analysis was performed for BI-EHEC and exhibited the highest similarity with *Escherichia* phage ESCO13. *Escherichia* phage anhysbys and ESCO13 of NCBI database were selected and served as a comparison genome for BRIG analysis.

Separate BRIG analyses were also carried out using BI-EHEC against lysogenic [20] and virulence genes [21]. However, it yielded no colored rings, which indicated no such genes present on BI-EHEC.

#### PHACTS and CARD

Analysis using PHACTS was performed to confirm that BI-EHEC have lytic life cycle properties. The average probability produced by PHACTS for BI-EHEC is 0.519 with 0.05 standard deviation, non-confidently declared lytic bacteriophage by PHACTS. However, PHACTS have a high confidence rate (up to 99%) in determining phage lifestyle. According to McNair et al. 2012 [18], there is a high chance that non-confident prediction would yield an exact result as predicted.

CARD analyze a molecular sequence for predicting resistome based on homology and SNP models with perfect and strict parameters yielded zero results, and it was changed to loose hits to accommodate, the complete result can be observed in Additional file 4: Table S3, with TriC as the highest result. The loose hits algorithm can detect in lower similarity (< 95%) and more distant homologs of ARGs genes [17]. However, it only yielded results with less than 95% similarity therefore it can mislabel unrelated genes as antibiotic-resistant genes.

### Discussion

Annotations using multiPhATE2 could annotate proteins which are necessary for the end of a lytic cycle or structural proteins (Table 1). It was also noted that among successfully annotated CDs, lysogenic genes were not able to be found.

The phage genome-packaging component itself consists of portal protein, small terminase and large terminase. Small terminase can be annotated using multiPhATE2 (Table 1), this protein is used to initiate genome packaging and regulating large terminase functions. Meanwhile, large terminase is important to cleave concatenated DNA molecules to initiate packaging mechanisms [22].

Assembly for phages is done separately for the head, the tail, and the long tail fibers before joining to form a mature phage [23, 24]. Both Tail fiber assembly (Tfa) and gpH were involved in the tail assembly. Tfa is a family of proteins play a role in folding phage fibers as chaperones and determining host range specificity. Tape important in measure protein gpH and determines the length of the phage tail [25, 26].

Putative T4-like lysozyme is a hydrolytic enzyme used to cleave peptidoglycan bonds, It is produced during the late stage of the lytic cycle when assembled phages are ready to be released to the environment. Lysin possesses two main domains, N-terminal functions as a catalytic domain while C-terminal serves as a binding domain that targets and binds to specific peptidoglycan ligands [3].

Tail fiber functions as a receptor-binding protein (RBP) in many bacteriophages. RBP plays a role in phage host recognition and its interaction with other phages of the same host. For T4-like phages, the C-terminal and N-terminal regions of tail fiber are important to determine the receptor specificity as well as host range [27, 28]. BI-EHEC tail fiber protein showed the closest with *Escherichia* phage ukendt with *E. coli* K-12 MG1655 as its host [29]. Similar genetic make-up might contribute to different phages having the same host range. It is beneficial to study and observe a variety of tail fiber genes to expand knowledge of the host range used in phage cocktails [14].

Resulting annotations and BRIG analysis showed no lysogenic and virulence genes on BI-EHEC. Lysogenic bacteriophages utilize integrase and excisionase, encoded by *int* and *xis*, to bind their DNA to the host’s [6, 20, 22]. For virulence genes, analysis was done to three major virulence genes of EHEC: *eae*, *lpf* and *stx*, which encode for intimin, long polar fimbriae (LPF) and Shiga toxin respectively. *Stx* is the major virulence determinant of EHEC. Meanwhile, intimin and LPF aid the attachment of EHEC to its host cell [21].

Analysis using CARD database was done to determine whether samples carry over ARGs from the host or not. A temperate phage has a higher probability of carrying host genes, at this state, phage integrated their genome into the host and depends on hosts favorable conditions, phages could co-existence (prophages embedded) inside the DNA of the hosts, and there are possibility where temperate phage could carry host genes [30]. The possibility to carry over ARGs is rarely found. It was suggested that up to 1000-fold uncommon for phages to transfer ARGs via transduction compared to other means [31].

Initial analysis using CARD was done using perfect and strict hits only parameters. However, this run yielded no results, which might indicate no ARGs present on BI-EHEC. Another analysis was conducted with the loose hits parameter, including hits with less than 95% homology matches across the database. It could be beneficial in detecting emerging threats. Unfortunately, it also produces homolog hits that might be unrelated to its function as ARGs. By including as many hits as possible, loose hits can detect unknown proteins which can potentially be a new antibiotic-resistant protein. However, it makes it less specific to detect actual antibiotic-resistant protein [17].

Analysis using loose hits showed TriC as the highest possible match for BI-EHEC. The suggested mechanism of action by TriC is antibiotic efflux [31]. While ARGs were found during CARD analysis with loose hits parameter, it was still possible to rule out the presence of ARGs. Another study found that some proteins might be mistakenly labelled as ARGs while using CARD. This finding is common with phage genomes containing several leftover DNA from host cells. It was also suggested to use only conservative parameters when using in silico analysis to achieve the best possible matches, implying that only perfect and strict hits results are eligible to be included [31].

From the data produced by multiPhATE2, BRIG, and CARD analysis, it could be concluded that BI-EHEC leans towards following a strictly lytic life cycle and ARGs were not found in bacteriophage genome.

### Conclusion

BI-EHEC were successfully annotated, including structural and lytic cycle-related genes. The phylogenetic tree of tail fiber protein and BRIG results showed that BI-EHEC were similar to phages of the same host in NCBI. There were no signs of virulence or lysogenic protein among annotated genes, and PHACTS analysis confirmed this notion further. CARD results indicate no ARGs present on BI-EHEC. It can be concluded that BI-EHEC is promising as candidate for food preservative.

#### Limitations

The lack of annotated genes (resulting in many hypothetical protein hits) on the database has proven to be the limitation of this research.

## Supplementary Information

**Additional file 1.** **Table S1** Full annotations of US-EHEC.

**Additional file 2.** **Figure S1** Genome map of BI-EHEC. Annotation was selected based on its role on lyric cycle and/or structural.

**Additional file 3.** **Table S2** List of tail fiber from NCBI database and its accession number.

**Additional file 4.** **Table S3** US-EHEC CARD results (highest to lowest best identities).

## Data Availability

The datasets used and/or analyzed during the current study are available from the corresponding author on reasonable request.

## Abbreviations

EHEC: Enterohemorrhagic *Escherichia coli*
ARGs: Antimicrobial resistance genes
NGS: Next generation sequencing
BLAST: Basic local alignment search tool
MEGA: Molecular evolutionary genetics analysis
BRIG: BLAST ring image generator
PHACTS: Phage classification tool set
CARD: Comprehensive antibiotic resistance database
NCBI: National Centre for Biotechnology Information
