# Supplementary material for: Genomic characterization of bacteriophage BI-EHEC infecting strains of Enterohemorrhagic Escherichia coli
Source: BMC Res Notes. 2021 Dec 20;14:459. doi: 10.1186/s13104-021-05881-5 (PMC8686590; doi:10.1186/s13104-021-05881-5)
Supplement: Supplementary file 1 — Additional file 1. Table S1 Full annotations of US-EHEC. [file 13104_2021_5881_MOESM1_ESM.docx]

Table S1 Full annotations of US-EHEC

| CDs | | | Annotations |
| --- | --- | --- | --- |
| us-ehec-1 | 89 | 238 | Putative exodeoxyribonuclease; *Sulfitobacter* phage pcb2047-A \| exodeoxyribonuclease; *Sulfitobacter* phage pcb2047-C |
| us-ehec-2 | 268 | 405 |  |
| us-ehec-3 | 451 | 582 | IFPLOHOB_00001 hypothetical protein \| DNHOGCFM_00017 hypothetical protein \| hypothetical protein phAPEC8_0018; *Escherichia* phage phAPEC8 \| HFBDACEP_00001 hypothetical protein \| JOODPJME_00217 hypothetical protein \| hypothetical protein; *Escherichia* phage phAPEC8 |
| us-ehec-4 | 614 | 952 |  |
| us-ehec-5 | 1012 | 1128 | HLAHOEIE_00049 hypothetical protein \| DNHOGCFM_00019 hypothetical protein \| hypothetical protein phAPEC8_0020; *Escherichia* phage phAPEC8 \| JIHLJMCN_00002 hypothetical protein \| BDMKCPGI_00002 hypothetical protein \| hypothetical protein; *Escherichia* phage phAPEC8 \| hypothetical protein phAPEC8_0021; *Escherichia* phage phAPEC8 |
| us-ehec-6 | 1125 | 1223 | Hypothetical protein phAPEC8_0021; *Escherichia* phage phAPEC8 \| JIHLJMCN_00003 hypothetical protein \| BDMKCPGI_00003 hypothetical protein \| DNHOGCFM_00020 hypothetical protein \| JOODPJME_00214 hypothetical protein \| hypothetical protein; *Escherichia* phage phAPEC8 \| hypothetical protein phAPEC8_0021; *Escherichia* phage phAPEC8 |
| us-ehec-7 | 1216 | 1446 | IFPLOHOB_00005 hypothetical protein \| BDMKCPGI_00004 hypothetical protein \| JOODPJME_00213 hypothetical protein \| JIHLJMCN_00004 hypothetical protein \| OAGBNOCD_00245 hypothetical protein \| hypothetical protein; *Escherichia* phage phAPEC8 |
| us-ehec-8 | 1459 | 1698 | HLAHOEIE_00051 hypothetical protein \| HFBDACEP_00006 hypothetical protein \| JOODPJME_00212 hypothetical protein \| OAGBNOCD_00246 hypothetical protein \| JIHLJMCN_00005 hypothetical protein |
| us-ehec-9 | 1695 | 2156 | OAGBNOCD_00247 hypothetical protein \| HLAHOEIE_00052 hypothetical protein \| DCKEIKPP_00259 hypothetical protein \| JLLHFBGM_00002 hypothetical protein \| PDNLIHIO_00002 hypothetical protein \| Phage protein; ACLAME_Phage_proteins_with_unknown_functions Phage_cyanophage  Phage_experimental; *Enterobacteria* phage phi92 |
| us-ehec-10 | 2149 | 2343 | HLAHOEIE_00053 hypothetical protein |
| us-ehec-11 | 2336 | 2791 | DNHOGCFM_00023 hypothetical protein \| hypothetical protein phAPEC8_0024; *Escherichia* phage phAPEC8 \| BDMKCPGI_00006 hypothetical protein \| JOODPJME_00211 hypothetical protein \| IFPLOHOB_00007 hypothetical protein \| hypothetical protein; *Escherichia* phage phAPEC8 |
| us-ehec-12 | 2797 | 2910 | HFBDACEP_00007 hypothetical protein \| JIHLJMCN_00006 hypothetical protein \| OAGBNOCD_00248 hypothetical protein \| BDMKCPGI_00006 hypothetical protein \| JOODPJME_00211 hypothetical protein |
| us-ehec-13 | 2940 | 3254 | HLAHOEIE_00055 hypothetical protein \| DNHOGCFM_00024 hypothetical protein \| hypothetical protein phAPEC8_0025; *Escherichia* phage phAPEC8 \| JOODPJME_00210 hypothetical protein \| BDMKCPGI_00007 hypothetical protein \| hypothetical protein; *Escherichia* phage phAPEC8 |
| us-ehec-14 | 3267 | 3704 | IFPLOHOB_00010 hypothetical protein \| JOODPJME_00209 hypothetical protein \| JIHLJMCN_00009 hypothetical protein \| HLAHOEIE_00056 hypothetical protein \| BDMKCPGI_00008 hypothetical protein \| hypothetical protein; *Escherichia* phage phAPEC8 |
| us-ehec-15 | 3732 | 4031 | HFBDACEP_00012 hypothetical protein \| JOODPJME_00207 hypothetical protein \| JIHLJMCN_00011 hypothetical protein \| HLAHOEIE_00058 hypothetical protein \| DNHOGCFM_00027 hypothetical protein \| hypothetical protein; *Escherichia* phage phAPEC8 \| hypothetical protein phAPEC8_0028; *Escherichia* phage phAPEC8 |
| us-ehec-16 | 4031 | 4264 | OAGBNOCD_00253 hypothetical protein |
| us-ehec-17 | 4277 | 4465 | OAGBNOCD_00253 hypothetical protein |
| us-ehec-18 | 4497 | 4751 | OAGBNOCD_00254 hypothetical protein \| HFBDACEP_00013 hypothetical protein \| JIHLJMCN_00012 hypothetical protein \| BDMKCPGI_00011 hypothetical protein \| IFPLOHOB_00013 hypothetical protein |
| us-ehec-19 | 4748 | 4966 | JIHLJMCN_00013 hypothetical protein \| HLAHOEIE_00060 hypothetical protein \| BDMKCPGI_00012 hypothetical protein \| IFPLOHOB_00013 hypothetical protein |
| us-ehec-20 | 4947 | 5138 | JIHLJMCN_00014 hypothetical protein \| HLAHOEIE_00061 hypothetical protein \| BDMKCPGI_00013 hypothetical protein |
| us-ehec-21 | 5148 | 5507 | JIHLJMCN_00015 hypothetical protein \| HLAHOEIE_00062 hypothetical protein \| DNHOGCFM_00028 hypothetical protein \| hypothetical protein phAPEC8_0029; *Escherichia* phage phAPEC8 \| IFPLOHOB_00014 hypothetical protein \| hypothetical protein; *Escherichia* phage phAPEC8, complete genome \| Tryptophan synthase alpha chain; *Desulfovibrio* *vulgaris* subsp. Vulgaris (strain DP4) \| Tryptophan synthase alpha chain; *Desulfovibrio vulgaris* (strain Hildenborough / ATCC 29579 / DSM 644 / NCIMB 8303) |
| us-ehec-22 | 5523 | 5699 | OAGBNOCD_00256 hypothetical protein \| HFBDACEP_00015 hypothetical protein \| JOODPJME_00205 hypothetical protein \| JIHLJMCN_00016 hypothetical protein \| HLAHOEIE_00063 hypothetical protein \| hypothetical protein; *Escherichia* phage phAPEC8 \| hypothetical protein phAPEC8_0030; *Escherichia* phage phAPEC8 |
| us-ehec-23 | 5700 | 6014 | JOODPJME_00204 hypothetical protein \| JIHLJMCN_00017 hypothetical protein \| OAGBNOCD_00257 hypothetical protein \| HLAHOEIE_00064 hypothetical protein \| DNHOGCFM_00030 hypothetical protein \| hypothetical protein; *Escherichia* phage phAPEC8 |
| us-ehec-24 | 6005 | 6205 | HFBDACEP_00017 hypothetical protein \| JOODPJME_00203 hypothetical protein \| JIHLJMCN_00018 hypothetical protein \| IFPLOHOB_00017 hypothetical protein \| OAGBNOCD_00258 hypothetical protein \| hypothetical protein; *Escherichia* phage phAPEC8 |
| us-ehec-25 | 6220 | 6408 | BDMKCPGI_00018 hypothetical protein \| OAGBNOCD_00259 hypothetical protein \| HFBDACEP_00018 hypothetical protein |
| us-ehec-26 | 6410 | 6631 | JOODPJME_00201 hypothetical protein \| BDMKCPGI_00019 hypothetical protein \| JIHLJMCN_00020 hypothetical protein \| HLAHOEIE_00067 hypothetical protein \| IFPLOHOB_00019 hypothetical protein \| hypothetical protein; *Escherichia* phage phAPEC8 |
| us-ehec-27 | 6634 | 6840 | HLAHOEIE_00068 hypothetical protein \| JOODPJME_00200 hypothetical protein \| HFBDACEP_00020 hypothetical protein \| DNHOGCFM_00034 hypothetical protein \| hypothetical protein phAPEC8_0035; *Escherichia* phage phAPEC8 \| hypothetical protein; *Escherichia* phage phAPEC8 |
| us-ehec-28 | 6937 | 7335 | DNHOGCFM_00035 hypothetical protein \| hypothetical protein phAPEC8_0036; *Escherichia* phage phAPEC8 \| hypothetical protein; *Escherichia* phage phAPEC8 |
| us-ehec-29 | 7346 | 7801 | JIHLJMCN_00024 hypothetical protein \| JOODPJME_00197 hypothetical protein \| HLAHOEIE_00071 hypothetical protein \| BDMKCPGI_00023 hypothetical protein \| HFBDACEP_00023 hypothetical protein \| hypothetical protein; *Escherichia* phage phAPEC8 |
| us-ehec-30 | 7951 | 8433 | JOODPJME_00196 hypothetical protein \| JIHLJMCN_00025 hypothetical protein \| HFBDACEP_00024 hypothetical protein \| IFPLOHOB_00024 hypothetical protein \| HLAHOEIE_00072 hypothetical protein \| hypothetical protein; *Escherichia* phage phAPEC8 |
| us-ehec-31 | 8430 | 8630 | JOODPJME_00195 hypothetical protein \| HFBDACEP_00025 hypothetical protein \| JIHLJMCN_00026 hypothetical protein \| DNHOGCFM_00039 hypothetical protein \| hypothetical protein phAPEC8_0040; *Escherichia* phage phAPEC8 \| hypothetical protein; *Escherichia* phage phAPEC8 \| hypothetical protein phAPEC8_0040; *Escherichia* phage phAPEC8 |
| us-ehec-32 | 8693 | 8965 | DNHOGCFM_00040 hypothetical protein \| hypothetical protein phAPEC8_0041; *Escherichia* phage phAPEC8 \| HFBDACEP_00026 hypothetical protein \| JIHLJMCN_00027 hypothetical protein \| JOODPJME_00194 hypothetical protein \| hypothetical protein; *Escherichia* phage phAPEC8 \| hypothetical protein phAPEC8_0041; *Escherichia* phage phAPEC8 |
| us-ehec-33 | 8931 | 9257 | JIHLJMCN_00028 hypothetical protein \| hypothetical protein; *Escherichia* phage phAPEC8 |
| us-ehec-34 | 9257 | 9484 | DNHOGCFM_00042 hypothetical protein \| hypothetical protein phAPEC8_0043; *Escherichia* phage phAPEC8 \| hypothetical protein; *Escherichia* phage phAPEC8 |
| us-ehec-35 | 9487 | 9699 | OAGBNOCD_00268 hypothetical protein \| JIHLJMCN_00030 hypothetical protein \| DNHOGCFM_00043 hypothetical protein \| hypothetical protein phAPEC8_0044; *Escherichia* phage phAPEC8 \| hypothetical protein; *Escherichia* phage phAPEC8 \| hypothetical protein phAPEC8_0044; *Escherichia* phage phAPEC8 |
| us-ehec-36 | 9696 | 10016 | JIHLJMCN_00031 hypothetical protein \| DNHOGCFM_00044 hypothetical protein \| hypothetical protein phAPEC8_0045; *Escherichia* phage phAPEC8 \| HFBDACEP_00029 hypothetical protein \| JOODPJME_00191 hypothetical protein \| hypothetical protein; *Escherichia* phage phAPEC8 |
| us-ehec-37 | 10071 | 10625 | JOODPJME_00190 hypothetical protein \| JIHLJMCN_00032 hypothetical protein \| DNHOGCFM_00045 hypothetical protein \| hypothetical protein phAPEC8_0046; *Escherichia* phage phAPEC8 \| BDMKCPGI_00030 hypothetical protein \| hypothetical protein; *Escherichia* phage phAPEC8 |
| us-ehec-38 | 10636 | 10935 | IFPLOHOB_00032 hypothetical protein \| DNHOGCFM_00046 hypothetical protein \| hypothetical protein phAPEC8_0047; *Escherichia* phage phAPEC8 \| JOODPJME_00189 hypothetical protein \| JIHLJMCN_00033 hypothetical protein \| hypothetical protein; *Escherichia* phage phAPEC8 |
| us-ehec-39 | 10946 | 11269 | OAGBNOCD_00272 hypothetical protein \| DNHOGCFM_00046 hypothetical protein \| hypothetical protein phAPEC8_0048; *Escherichia* phage phAPEC8 \| HLAHOEIE_00080 hypothetical protein \| JOODPJME_00188 hypothetical protein \| hypothetical protein; *Escherichia* phage phAPEC8 \| hypothetical protein; *Yersinia* phage phid1 \| Phage protein; ACLAME_Phage_proteins_with_unknown_functions Phage_cyanophage Phage_experimental; T4-like viruses *Enterobacteria* phage RB51 \| Uncharacterized 8.5 kda protein in tk-vs intergenic region ; *Enterobacteria* phage T4 |
| us-ehec-40 | 11270 | 11482 | JOODPJME_00187 hypothetical protein \| JIHLJMCN_00035 hypothetical protein \| OAGBNOCD_00273 hypothetical protein] \| HLAHOEIE_00081 hypothetical protein \| BDMKCPGI_00033 hypothetical protein \| hypothetical protein; *Escherichia* phage phAPEC8 \| hypothetical protein phAPEC8_0049 ; *Escherichia* phage phAPEC8 |
| us-ehec-41 | 11553 | 12224 | OAGBNOCD_00274 hypothetical protein \| DNHOGCFM_00049 hypothetical protein \| hypothetical protein phAPEC8_0050; *Escherichia* phage phAPEC8 \| JOODPJME_00186 hypothetical protein \| HFBDACEP_00036 hypothetical protein \| hypothetical protein; *Escherichia* phage phAPEC8 |
| us-ehec-42 | 12233 | 12643 | OAGBNOCD_00275 hypothetical protein \| HLAHOEIE_00083 hypothetical protein \| JIHLJMCN_00037 hypothetical protein \| JOODPJME_00185 hypothetical protein \| HFBDACEP_00037 hypothetical protein \| hypothetical protein; *Escherichia* phage phAPEC8 |
| us-ehec-43 | 12633 | 12941 | JOODPJME_00184 hypothetical protein \| JIHLJMCN_00038 hypothetical protein \| HFBDACEP_00038 hypothetical protein \| DNHOGCFM_00051 hypothetical protein \| hypothetical protein phAPEC8_0052; *Escherichia* phage phAPEC8 \| hypothetical protein; *Escherichia* phage phAPEC8 |
| us-ehec-44 | 12999 | 13271 | JOODPJME_00183 hypothetical protein \| DMKCPGI_00037 hypothetical protein \| HFBDACEP_00039 hypothetical protein \| IFPLOHOB_00038 hypothetical protein \| DNHOGCFM_00052 hypothetical protein \| hypothetical protein; *Escherichia* phage phAPEC8 |
| us-ehec-45 | 13276 | 13887 | HLAHOEIE_00086 hypothetical protein \| DNHOGCFM_00053 hypothetical protein \| hypothetical protein phAPEC8_0054; *Escherichia* phage phAPEC8 \| IFPLOHOB_00039 hypothetical protein \| JOODPJME_00182 hypothetical protein \| hypothetical protein; *Escherichia* phage phAPEC8 \| hypothetical protein phAPEC8_0054; *Escherichia* phage phAPEC8 |
| us-ehec-46 | 13939 | 14277 | DNHOGCFM_00054 hypothetical protein \| hypothetical protein phAPEC8_0055; *Escherichia* phage phAPEC8 \| BDMKCPGI_00039 hypothetical protein \| JOODPJME_00181 hypothetical protein \| JIHLJMCN_00041 hypothetical protein \| hypothetical protein; *Escherichia* phage phAPEC8 \| hypothetical protein; *Escherichia* phage phAPEC8 |
| us-ehec-47 | 14264 | 14596 | JOODPJME_00179 hypothetical protein \| HFBDACEP_00043 hypothetical protein \| BDMKCPGI_00041 hypothetical protein \| JIHLJMCN_00043 hypothetical protein \| IFPLOHOB_00041 hypothetical protein \| hypothetical protein; *Escherichia* phage phAPEC8 |
| us-ehec-48 | 14614 | 15033 | \| |
| us-ehec-49 | 15068 | 15262 | \| |
| us-ehec-50 | 15538 | 15663 | JOODPJME_00177 hypothetical protein \| JIHLJMCN_00045 hypothetical protein \| BDMKCPGI_00043 hypothetical protein \| HFBDACEP_00045 hypothetical protein \| DNHOGCFM_00058 hypothetical protein \| hypothetical protein; *Escherichia* phage phAPEC8 |
| us-ehec-51 | 15596 | 15811 | JOODPJME_00176 hypothetical protein \| JIHLJMCN_00046 hypothetical protein \| OAGBNOCD_00001 hypothetical protein \| HLAHOEIE_00092 hypothetical protein \| BDMKCPGI_00044 hypothetical protein \| hypothetical protein; *Escherichia* phage phAPEC8 \| hypothetical protein Syn1_159; *Prochlorococcus* phage Syn1 |
| us-ehec-52 | 15825 | 16043 | JOODPJME_00175 hypothetical protein \| JIHLJMCN_00047 hypothetical protein \| OAGBNOCD_00002 hypothetical protein \| BDMKCPGI_00045 hypothetical protein \| HFBDACEP_00047 hypothetical protein \| hypothetical protein; *Escherichia* phage phAPEC8 |
| us-ehec-53 | 16046 | 16243 | JOODPJME_00174 hypothetical protein \| JIHLJMCN_00048 hypothetical protein \| BDMKCPGI_00046 hypothetical protein \| HFBDACEP_00048 hypothetical protein \| IFPLOHOB_00046 hypothetical protein \| hypothetical protein; *Escherichia* phage phAPEC8 |
| us-ehec-54 | 16227 | 16676 | JOODPJME_00173 hypothetical protein \| OAGBNOCD_00004 hypothetical protein \| HLAHOEIE_00094 hypothetical protein \| BDMKCPGI_00047 hypothetical protein \| HFBDACEP_00049 hypothetical protein \| hypothetical protein; *Escherichia* phage phAPEC8 \| hypothetical protein phAPEC8_0064; *Escherichia* phage phAPEC8 |
| us-ehec-55 | 16676 | 16840 | OAGBNOCD_00005 hypothetical protein \| HLAHOEIE_00095 hypothetical protein \| DNHOGCFM_00063 hypothetical protein \| hypothetical protein phAPEC8_0065; *Escherichia* phage phAPEC8 \| JIHLJMCN_00050 hypothetical protein \| hypothetical protein; *Escherichia* phage phAPEC8 |
| us-ehec-56 | 16837 | 17100 | JOODPJME_00171 hypothetical protein \| JIHLJMCN_00051 hypothetical protein \| OAGBNOCD_00006 hypothetical protein \| BDMKCPGI_00049 hypothetical protein \| HFBDACEP_00051 hypothetical protein \| hypothetical protein; *Escherichia* phage phAPEC8 |
| us-ehec-57 | 17109 | 17717 | JOODPJME_00170 hypothetical protein \| JIHLJMCN_00052 hypothetical protein \| OAGBNOCD_00007 hypothetical protein \| HLAHOEIE_00097 hypothetical protein \| BDMKCPGI_00050 hypothetical protein \| hypothetical protein; *Escherichia* phage phAPEC8 \| Zinc finger protein 778; Homo sapiens |
| us-ehec-58 | 17727 | 17828 | JOODPJME_00169 hypothetical protein \| JIHLJMCN_00053 hypothetical protein \| OAGBNOCD_00008 hypothetical protein \| HLAHOEIE_00098 hypothetical protein \| BDMKCPGI_00051 hypothetical protein \| hypothetical protein; *Escherichia* phage phAPEC8 \| hypothetical protein phAPEC8_0068; *Escherichia* phage phAPEC8 |
| us-ehec-59 | 17881 | 18054 | JOODPJME_00168 hypothetical protein \| JIHLJMCN_00054 hypothetical protein \| BDMKCPGI_00052 hypothetical protein \| HFBDACEP_00054 hypothetical protein \| DNHOGCFM_00067 hypothetical protein \| hypothetical protein; *Escherichia* phage phAPEC8 \| major capsid protein; *Bacillus* phage VMY22 |
| us-ehec-60 | 18067 | 18348 | JOODPJME_00167 hypothetical protein \| JIHLJMCN_00055 hypothetical protein \| HLAHOEIE_00100 hypothetical protein \| BDMKCPGI_00053 hypothetical protein \| HFBDACEP_00055 hypothetical protein \| hypothetical protein; *Escherichia* phage phapec |
| us-ehec-61 | 18440 | 18670 | JIHLJMCN_00056 hypothetical protein \| OAGBNOCD_00011 hypothetical protein \| HLAHOEIE_00101 hypothetical protein \| HFBDACEP_00056 hypothetical protein \| DNHOGCFM_00069 hypothetical protein \| hypothetical protein; *Escherichia* phage phAPEC8 |
| us-ehec-62 | 18740 | 19012 | JOODPJME_00165 NAD-dependent protein deacylase \| IFPLOHOB_00055 NAD-dependent protein deacylase \| HFBDACEP_00057 NAD-dependent protein deacylase \| HLAHOEIE_00102 NAD-dependent protein deacylase \| NAD-dependent protein deacylase \| NAD-dependent protein deacetylase of SIR2 family; *Enterobacteria* phage phi92 \| putative Sir2-like transferase; *Enterobacteria* phage ECGD1 \| Phi92_gp038; *Enterobacteria* phage phi92 |
| us-ehec-63 | 19056 | 19439 | JIHLJMCN_00057 NAD-dependent protein deacylase \| JOODPJME_00165 NAD-dependent protein deacylase \| IFPLOHOB_00055 NAD-dependent protein deacylase \| HLAHOEIE_00102 NAD-dependent protein deacylase \| HFBDACEP_00057 NAD-dependent protein deacylase \| NAD-dependent protein deacetylase of SIR2 family; *Enterobacteria* phage phi92 \| putative Sir2-like transferase; *Enterobacteria* phage ECGD1 \| Phi92_gp038; *Enterobacteria* phage phi92 |
| us-ehec-64 | 19453 | 19872 | JOODPJME_00164 hypothetical protein \| JIHLJMCN_00058 hypothetical protein \| HFBDACEP_00058 hypothetical protein \| IFPLOHOB_00056 hypothetical protein \| HLAHOEIE_00103 hypothetical protein \| hypothetical protein; *Escherichia* phage phAPEC8 |
| us-ehec-65 | 19857 | 20342 | JIHLJMCN_00059 hypothetical protein \| OAGBNOCD_00014 hypothetical protein \| HFBDACEP_00059 hypothetical protein \| HLAHOEIE_00104 hypothetical protein \| DNHOGCFM_00072 hypothetical protein \| hypothetical protein; *Escherichia* phage phAPEC8 \| hypothetical protein phAPEC8_0074; *Escherichia* phage phAPEC8 |
| us-ehec-66 | 20401 | 21054 | JOODPJME_00162 hypothetical protein \| JIHLJMCN_00060 hypothetical protein \| OAGBNOCD_00015 hypothetical protein \| HLAHOEIE_00105 hypothetical protein \| BDMKCPGI_00058 hypothetical protein \| hypothetical protein; *Escherichia* phage phAPEC8 \| hypothetical protein phAPEC8_0075; *Escherichia* phage phAPEC8 |
| us-ehec-67 | 21054 | 21737 | DNHOGCFM_00074 hypothetical protein \| hypothetical protein phAPEC8_0076; *Escherichia* phage phAPEC8 \| JOODPJME_00161 hypothetical protein \| JIHLJMCN_00061 hypothetical protein \| OAGBNOCD_00016 hypothetical protein \| hypothetical protein; *Escherichia* phage phAPEC8 \| ORF005; Staphylococcus phage 2638A |
| us-ehec-68 | 21730 | 22125 | OAGBNOCD_00017 hypothetical protein \| JOODPJME_00160 hypothetical protein \| HLAHOEIE_00107 hypothetical protein \| IFPLOHOB_00060 hypothetical protein \| DNHOGCFM_00075 hypothetical protein \| hypothetical protein; *Escherichia* phage phAPEC8 |
| us-ehec-69 | 22127 | 23068 | JOODPJME_00159 hypothetical protein \| JIHLJMCN_00063 hypothetical protein \| OAGBNOCD_00018 hypothetical protein \| HLAHOEIE_00108 hypothetical protein \| BDMKCPGI_00061 hypothetical protein \| hypothetical protein; *Escherichia* phage phAPEC8 \| hypothetical protein phAPEC8_0078; *Escherichia* phage phAPEC8 \| alpha-mannosidase (EC 3.2.1.24) |
| us-ehec-70 | 23159 | 23431 | DNHOGCFM_00077 hypothetical protein \| hypothetical protein phAPEC8_0079; *Escherichia* phage phAPEC8 \| HLAHOEIE_00109 hypothetical protein \| HFBDACEP_00064 hypothetical protein \| BDMKCPGI_00062 hypothetical protein \| hypothetical protein; *Escherichia* phage phAPEC8 \| hypothetical protein phAPEC8_0079; *Escherichia* phage phAPEC8 |
| us-ehec-71 | 23441 | 23659 | OAGBNOCD_00020 hypothetical protein \| HLAHOEIE_00110 hypothetical protein \| HFBDACEP_00065 hypothetical protein \| DNHOGCFM_00078 hypothetical protein \| hypothetical protein phAPEC8_0080; *Escherichia* phage phAPEC8 \| hypothetical protein; *Escherichia* phage phAPEC8 \| Uncharacterized 10.2 kda protein in regV-denV intergenic region; *Enterobacteria* phage T4 \| hypothetical protein phAPEC8_0080; *Escherichia* phage phAPEC8 |
| us-ehec-72 | 23659 | 23928 | JOODPJME_00156 hypothetical protein \| JIHLJMCN_00066 hypothetical protein \| HLAHOEIE_00111 hypothetical protein \| BDMKCPGI_00064 hypothetical protein \| IFPLOHOB_00064 hypothetical protein \| hypothetical protein; *Escherichia* phage phAPEC8 \| hypothetical protein phAPEC8_0081; *Escherichia* phage phAPEC8 |
| us-ehec-73 | 23940 | 24149 | JOODPJME_00155 hypothetical protein \| JIHLJMCN_00067 hypothetical protein \| OAGBNOCD_00022 hypothetical protein \| HLAHOEIE_00112 hypothetical protein \| HFBDACEP_00067 hypothetical protein \| hypothetical protein; *Escherichia* phage phAPEC8 \| hypothetical protein phAPEC8_0082; *Escherichia* phage phAPEC8 |
| us-ehec-74 | 24121 | 24411 | HLAHOEIE_00113 hypothetical protein \| JOODPJME_00154 hypothetical protein \| JIHLJMCN_00068 hypothetical protein \| BDMKCPGI_00066 hypothetical protein \| HFBDACEP_00068 hypothetical protein \| hypothetical protein; *Escherichia* phage phAPEC8 |
| us-ehec-75 | 24390 | 24839 | JOODPJME_00153 hypothetical protein \| JIHLJMCN_00069 hypothetical protein \| OAGBNOCD_00024 hypothetical protein \| HFBDACEP_00069 hypothetical protein \| DNHOGCFM_00082 hypothetical protein \| hypothetical protein; *Escherichia* phage phAPEC8 \| hypothetical protein phAPEC8_0084; *Escherichia* phage phAPEC8 |
| us-ehec-76 | 24892 | 25473 | JOODPJME_00152 hypothetical protein \| JIHLJMCN_00070 hypothetical protein \| OAGBNOCD_00025 hypothetical protein \| HLAHOEIE_00115 hypothetical protein \| BDMKCPGI_00068 hypothetical protein \| hypothetical protein; *Escherichia* phage phAPEC8 |
| us-ehec-77 | 25481 | 25789 | JOODPJME_00151 Serine/threonine-protein phosphatase 2 \| JIHLJMCN_00071 Serine/threonine-protein phosphatase 2 \| OAGBNOCD_00026 Serine/threonine-protein phosphatase 2 \| BDMKCPGI_00069 Serine/threonine-protein phosphatase 2 \| HFBDACEP_00071 Serine/threonine-protein phosphatase 2 \| putative serine/threonine protein phosphatase; *Escherichia* phage phAPEC8 |
| us-ehec-78 | 25789 | 26532 | JOODPJME_00150 hypothetical protein \| OAGBNOCD_00027 hypothetical protein \| HLAHOEIE_00117 hypothetical protein \| BDMKCPGI_00070 hypothetical protein \| HFBDACEP_00072 hypothetical protein \| hypothetical protein; *Escherichia* phage phAPEC8 |
| us-ehec-79 | 26529 | 26858 | DNHOGCFM_00086 hypothetical protein \| hypothetical protein phAPEC8_0088; *Escherichia* phage phAPEC8 \| JOODPJME_00149 hypothetical protein \| JIHLJMCN_00073 hypothetical protein \| HLAHOEIE_00118 hypothetical protein \| hypothetical protein; *Escherichia* phage phAPEC8 |
| us-ehec-80 | 26869 | 27441 | JOODPJME_00148 hypothetical protein \| JIHLJMCN_00074 hypothetical protein \| OAGBNOCD_00029 hypothetical protein \| HLAHOEIE_00119 hypothetical protein \| BDMKCPGI_00072 hypothetical protein \| hypothetical protein; *Escherichia* phage phAPEC8 \| hypothetical protein JWAP_00025; *Achromobacter* phage 83-24 |
| us-ehec-81 | 27456 | 27650 | JOODPJME_00147 hypothetical protein \| JIHLJMCN_00075 hypothetical protein \| HLAHOEIE_00120 hypothetical protein \| BDMKCPGI_00073 hypothetical protein \| HFBDACEP_00075 hypothetical protein \| hypothetical protein; *Escherichia* phage phAPEC8 |
| us-ehec-82 | 27696 | 27899 | JOODPJME_00146 hypothetical protein \| JIHLJMCN_00076 hypothetical protein \| HLAHOEIE_00121 hypothetical protein \| BDMKCPGI_00074 hypothetical protein \| HFBDACEP_00076 hypothetical protein \| hypothetical protein; *Escherichia* phage phAPEC8 |
| us-ehec-83 | 27899 | 28135 | JOODPJME_00145 hypothetical protein \| JIHLJMCN_00077 hypothetical protein \| OAGBNOCD_00032 hypothetical protein \| HLAHOEIE_00122 hypothetical protein \| BDMKCPGI_00075 hypothetical protein \| hypothetical protein; *Escherichia* phage phAPEC8 \| hypothetical protein phAPEC8_0092; *Escherichia* phage phAPEC8 |
| us-ehec-84 | 28148 | 28657 | JOODPJME_00144 hypothetical protein \| JIHLJMCN_00078 hypothetical protein \| HLAHOEIE_00123 hypothetical protein \| BDMKCPGI_00076 hypothetical protein \| HFBDACEP_00078 hypothetical protein \| hypothetical protein; *Escherichia* phage phAPEC8 |
| us-ehec-85 | 28641 | 29075 | JOODPJME_00143 ATP-dependent protease subunit hslv \| JIHLJMCN_00079 ATP-dependent protease subunit HslV \| OAGBNOCD_00034 ATP-dependent protease subunit HslV \| HLAHOEIE_00124 ATP-dependent protease subunit HslV \| BDMKCPGI_00077 ATP-dependent protease subunit HslV \| hypothetical protein; *Escherichia* phage phAPEC8 \| hypothetical protein phAPEC8_0094; *Escherichia* phage phAPEC8 |
| us-ehec-86 | 29121 | 29813 | JOODPJME_00142 hypothetical protein \| JIHLJMCN_00080 hypothetical protein \| OAGBNOCD_00035 hypothetical protein \| HLAHOEIE_00125 hypothetical protein \| BDMKCPGI_00078 hypothetical protein \| hypothetical protein; *Escherichia* phage phAPEC8 |
| us-ehec-87 | 29813 | 30313 | JOODPJME_00141 PhoH-like protein \| JIHLJMCN_00081 PhoH-like protein \| OAGBNOCD_00036 PhoH -like protein \| HLAHOEIE_00126 PhoH -like protein \| BDMKCPGI_00079 PhoH -like protein \| putative phoh family protein; *Escherichia* phage phAPEC8 \| putative PhoH family protein; *Escherichia* phage phAPEC8 |
| us-ehec-88 | 30349 | 31107 | JIHLJMCN_00082 hypothetical protein \| HLAHOEIE_00127 hypothetical protein \| BDMKCPGI_00080 hypothetical protein \| HFBDACEP_00082 hypothetical protein \| IFPLOHOB_00080 hypothetical protein \| putative bacteriophage T4-like lysozyme; *Escherichia* phage phAPEC8, complete genome \| putative bacteriophage T4-like lysozyme; *Escherichia* phage phAPEC8 \| lysozyme (EC 3.2.1.17) \| lysozyme (EC 3.2.1.17) \| lysozyme (EC 3.2.1.17) \| lysozyme (EC 3.2.1.17) \| lysozyme (EC 3.2.1.17) |
| us-ehec-89 | 31145 | 31645 | JOODPJME_00139 hypothetical protein \| JIHLJMCN_00083 hypothetical protein \| OAGBNOCD_00038 hypothetical protein \| HLAHOEIE_00128 hypothetical protein \| BDMKCPGI_00081 hypothetical protein \| hypothetical protein; *Escherichia* phage phAPEC8 |
| us-ehec-90 | 31653 | 31802 | JOODPJME_00138 hypothetical protein \| JIHLJMCN_00084 hypothetical protein \| OAGBNOCD_00039 hypothetical protein \| HLAHOEIE_00129 hypothetical protein \| HFBDACEP_00084 hypothetical protein \| putative glutaredoxin ; *Escherichia* phage phAPEC8 \| putative glutaredoxin 1; *Escherichia* phage phAPEC8 |
| us-ehec-91 | 31808 | 32068 | HLAHOEIE_00130 dTDP-4-dehydrorhamnose reductase \| OAGBNOCD_00040 dTDP-4-dehydrorhamnose reductase \| BDMKCPGI_00083 dTDP-4-dehydrorhamnose reductase \| JOODPJME_00137 dTDP-4-dehydrorhamnose reductase \| DNHOGCFM_00098 dTDP-4-dehydrorhamnose reductase \| putative dTDP-4-dehydrorhamnose reductase ; *Escherichia* phage phAPEC8 |
| us-ehec-92 | 32068 | 32910 | JOODPJME_00136 dTDP-4-dehydrorhamnose 3,5-epimerase \| JIHLJMCN_00086 dTDP-4-dehydrorhamnose 3,5-epimerase \| IFPLOHOB_00084 dTDP-4-dehydrorhamnose 3,5-epimerase \| DNHOGCFM_00099 dTDP-4-dehydrorhamnose 3,5-epimerase \| putative dTDP-4-dehydrorhamnose 3,5-epimerase; *Escherichia* phage phAPEC8 \| putative dTDP-4-dehydrorhamnose 3,5-epimerase; *Escherichia* phage phAPEC8 \| putative dTDP-4-dehydrorhamnose 3,5-epimerase; *Escherichia* phage phAPEC8] |
| us-ehec-93 | 32904 | 33470 | HLAHOEIE_00132 Glucose-1-phosphate thymidylyltransferase 2 \| IFPLOHOB_00085 Glucose-1-phosphate thymidylyltransferase 2 \| JIHLJMCN_00087 Glucose-1-phosphate thymidylyltransferase 2 \| BDMKCPGI_00085 Glucose-1-phosphate thymidylyltransferase 2 \| HFBDACEP_00087 Glucose-1-phosphate thymidylyltransferase 2 \| putative glucose-1-phosphate thymidylyltransferase; *Escherichia* phage phAPEC8 \| Glucose-1-phosphate thymidylyltransferase; *Neisseria meningitidis* serogroup B (strain MC58) \| Glucose-1-phosphate thymidylyltransferase; *Neisseria meningitidis* serogroup A / serotype 4A (strain DSM 15465 /  Z2491) \| Glucose-1-phosphate thymidylyltransferase ; Neisseria gonorrhoeae \| Glucose-1-phosphate thymidylyltransferase; *Streptococcus mutans* serotype c (strain ATCC 700610 / UA159) \| Glucose-1-phosphate thymidylyltransferase; *Yersinia* enterocolitica \| putative glucose-1-phosphate thymidylyltransferase; *Escherichia* phage phAPEC8 |
| us-ehec-94 | 33467 | 34345 | HLAHOEIE_00133 dTDP-glucose 4,6-dehydratase \| HFBDACEP_00088 dTDP-glucose 4,6-dehydratase \| IFPLOHOB_00086 dTDP-glucose 4,6-dehydratase \| BDMKCPGI_00086 dTDP-glucose 4,6-dehydratase \| DNHOGCFM_00101 dTDP-glucose 4,6-dehydratase \| putative dTDP-glucose 4,6-dehydratase; *Escherichia* phage phAPEC8 \| putative dTDP-glucose 4,6-dehydratase; *Escherichia* phage phAPEC8 |
| us-ehec-95 | 34354 | 35346 | OAGBNOCD_00042 hypothetical protein \| JIHLJMCN_00089 hypothetical protein \| DNHOGCFM_00102 hypothetical protein \| IFPLOHOB_00087 hypothetical protein \| hypothetical protein phAPEC8_00104; *Escherichia* phage phAPEC8 \| hypothetical protein; *Escherichia* phage phAPEC8 \| Phage protein; ACLAME_Phage_proteins_with_unknown_functions Phage_cyanophage Phage_experimental; *Enterobacteria* phage phi92 |
| us-ehec-96 | 35356 | 35682 | JIHLJMCN_00090 hypothetical protein \| HLAHOEIE_00135 hypothetical protein \| HFBDACEP_00090 hypothetical protein \| IFPLOHOB_00088 hypothetical protein \| OAGBNOCD_00043 hypothetical protein \| hypothetical protein; *Escherichia* phage phAPEC8 |
| us-ehec-97 | 35672 | 35836 | OAGBNOCD_00044 hypothetical protein \| DNHOGCFM_00104 hypothetical protein \| hypothetical protein phAPEC8_00106; *Escherichia* phage phAPEC8 \| JOODPJME_00131 hypothetical protein \| JIHLJMCN_00091 hypothetical protein \| hypothetical protein; *Escherichia* phage phAPEC8 \| Chaperone protein dnaj; Thio*Bacillus* denitrificans (strain ATCC 25259) |
| us-ehec-98 | 35826 | 35972 | JOODPJME_00130 hypothetical protein \| OAGBNOCD_00045 hypothetical protein \| DNHOGCFM_00105 hypothetical protein \| hypothetical protein phAPEC8_00107; *Escherichia* phage phAPEC8 \| JIHLJMCN_00092 hypothetical protein \| hypothetical protein' *Escherichia* phage phAPEC8 \| hypothetical protein phAPEC8_00107; *Escherichia* phage phAPEC8 |
| us-ehec-99 | 35972 | 36181 | JIHLJMCN_00093 Ribonucleoside-diphosphate reductase 1 subunit beta \| HLAHOEIE_00138 Ribonucleoside-diphosphate reductase 1 subunit beta \| BDMKCPGI_00091 Ribonucleoside-diphosphate reductase 1 subunit beta \| HFBDACEP_00093 Ribonucleoside-diphosphate reductase 1 subunit beta \| IFPLOHOB_00091 Ribonucleoside-diphosphate reductase 1 subunit beta \| Putative ribonucleotide reductase of class Ia (aerobic) beta subunit; *Escherichia* phage phAPEC8 \| Putative ribonucleotide reductase of class Ia (aerobic) beta subunit; *Escherichia* phage phAPEC8 |
| us-ehec-100 | 36181 | 37266 | JIHLJMCN_00094 Ribonucleoside-diphosphate reductase 1 subunit alpha \| IFPLOHOB_00092 Ribonucleoside-diphosphate reductase 1 subunit alpha \| DNHOGCFM_00108 Ribonucleoside-diphosphate reductase 1 subunit alpha \| Putative ribonucleotide reductase of class Ia (aerobic) alpha subunit; *Escherichia* phage phAPEC8 \| HLAHOEIE_00139 Ribonucleoside-diphosphate reductase 1 subunit alpha \| Putative ribonucleotide reductase of class Ia (aerobic) alpha subunit; *Escherichia* phage phAPEC8 \| Putative ribonucleotide reductase of class Ia (aerobic) alpha subunit; *Escherichia* phage phAPEC8 |
| us-ehec-101 | 37311 | 39551 | JOODPJME_00127 hypothetical protein \| JIHLJMCN_00095 hypothetical protein \| OAGBNOCD_00049 hypothetical protein \| HLAHOEIE_00140 hypothetical protein \| HFBDACEP_00096 hypothetical protein \| hypothetical protein; *Escherichia* phage phAPEC8 \| Phage protein; ACLAME_Phage_proteins_with_unknown_functions Phage_cyanophage Phage_experimental; *Enterobacteria* phage phi92 |
| us-ehec-102 | 39568 | 39822 | JOODPJME_00126 hypothetical protein \| JIHLJMCN_00096 hypothetical protein \| HLAHOEIE_00141 hypothetical protein \| BDMKCPGI_00096 hypothetical protein \| HFBDACEP_00097 hypothetical protein \| hypothetical protein; *Escherichia* phage phAPEC8 |
| us-ehec-103 | 39819 | 40229 | BDMKCPGI_00097 hypothetical protein \| OAGBNOCD_00051 hypothetical protein \| JIHLJMCN_00097 hypothetical protein \| HLAHOEIE_00142 hypothetical protein \| IFPLOHOB_00095 hypothetical protein \| putative thymidylate synthase; *Escherichia* phage phAPEC8 \| putative thymidylate synthase; *Escherichia* phage phAPEC8 |
| us-ehec-104 | 40328 | 41326 | JOODPJME_00124 hypothetical protein \| JIHLJMCN_00098 hypothetical protein \| HLAHOEIE_00143 hypothetical protein \| BDMKCPGI_00098 hypothetical protein \| HFBDACEP_00099 hypothetical protein \| hypothetical protein; *Escherichia* phage phAPEC8 \| hypothetical protein phAPEC8_00114; *Escherichia* phage phAPEC8 |
| us-ehec-105 | 41336 | 41944 | JOODPJME_00123 hypothetical protein \| JIHLJMCN_00099 hypothetical protein \| HLAHOEIE_00144 hypothetical protein \| IFPLOHOB_00097 hypothetical protein \| BDMKCPGI_00099 hypothetical protein \| hypothetical protein; *Escherichia* phage phAPEC8 \| hypothetical protein phAPEC8_00115; *Escherichia* phage phAPEC8 |
| us-ehec-106 | 41981 | 42535 | JOODPJME_00122 hypothetical protein \| JIHLJMCN_00100 hypothetical protein \| OAGBNOCD_00054 hypothetical protein \| HLAHOEIE_00145 hypothetical protein \| BDMKCPGI_00100 hypothetical protein \| hypothetical protein; *Escherichia* phage phAPEC8 \| hypothetical protein phAPEC8_00116; *Escherichia* phage phAPEC8 |
| us-ehec-107 | 42546 | 42740 | JOODPJME_00121 hypothetical protein \| OAGBNOCD_00055 hypothetical protein \| BDMKCPGI_00101 hypothetical protein \| HFBDACEP_00102 hypothetical protein \| DNHOGCFM_00115 hypothetical protein \| hypothetical protein; *Escherichia* phage phAPEC8 |
| us-ehec-108 | 42724 | 43125 | OAGBNOCD_00056 hypothetical protein \| JIHLJMCN_00102 hypothetical protein \| HLAHOEIE_00147 hypothetical protein \| HFBDACEP_00103 hypothetical protein \| IFPLOHOB_00100 hypothetical protein \| hypothetical protein; *Escherichia* phage phAPEC8 |
| us-ehec-109 | 43118 | 43345 | JIHLJMCN_00103 hypothetical protein \| HLAHOEIE_00148 hypothetical protein \| HFBDACEP_00104 hypothetical protein \| IFPLOHOB_00101 hypothetical protein \| JOODPJME_00119 hypothetical protein \| hypothetical protein; *Escherichia* phage phAPEC8 \| hypothetical protein phAPEC8_00119; *Escherichia* phage phAPEC8 |
| us-ehec-110 | 43432 | 44391 | JOODPJME_00118 hypothetical protein \| JIHLJMCN_00104 hypothetical protein \| OAGBNOCD_00058 hypothetical protein \| HLAHOEIE_00149 hypothetical protein \| BDMKCPGI_00104 hypothetical protein \| putative exonuclease; *Escherichia* phage phAPEC8 \| putative exonuclease; *Escherichia* phage phAPEC8 |
| us-ehec-111 | 44388 | 45077 | Hypothetical protein phAPEC8_00121 ; *Escherichia* phage phAPEC8 \| Hypothetical protein; *Escherichia* phage phAPEC8 |
| us-ehec-112 | 45155 | 45277 |  |
| us-ehec-113 | 45287 | 45379 | JOODPJME_00117 hypothetical protein \| JIHLJMCN_00105 hypothetical protein \| HLAHOEIE_00150 hypothetical protein \| HFBDACEP_00106 hypothetical protein \| DNHOGCFM_00119 hypothetical protein \| hypothetical protein; *Escherichia* phage phAPEC8 \| hypothetical protein phAPEC8_00122; *Escherichia* phage phAPEC8 |
| us-ehec-114 | 45519 | 45728 | JOODPJME_00116 Recombination-associated protein RdgC \| JIHLJMCN_00106 Recombination-associated protein RdgC \| HLAHOEIE_00151 Recombination-associated protein RdgC \| HFBDACEP_00107 Recombination-associated protein RdgC \| IFPLOHOB_00104 Recombination-associated protein RdgC \| Putative exonuclease RdgC; *Escherichia* phage phAPEC8 \| Putative exonuclease RdgC; *Escherichia* phage phAPEC8 |
| us-ehec-115 | 45801 | 46679 | JOODPJME_00115 hypothetical protein \| JIHLJMCN_00107 hypothetical protein \| HLAHOEIE_00152 hypothetical protein \| HFBDACEP_00108 hypothetical protein \| DNHOGCFM_00121 hypothetical protein \| hypothetical protein; *Escherichia* phage phAPEC8 \| hypothetical protein phAPEC8_00124; *Escherichia* phage phAPEC8 |
| us-ehec-116 | 46736 | 47461 | JOODPJME_00114 hypothetical protein \| HLAHOEIE_00153 hypothetical protein \| BDMKCPGI_00108 hypothetical protein \| HFBDACEP_00109 hypothetical protein \| IFPLOHOB_00106 hypothetical protein \| hypothetical protein; *Escherichia* phage phAPEC8 |
| us-ehec-117 | 47471 | 47653 | CMAOGEGD_00005 hypothetical protein \| YP_003104803.1 L2 ; *Francolinus leucoscepus* papillomavirus 1 |
| us-ehec-118 | 47703 | 47792 | JIHLJMCN_00110 hypothetical protein \| OAGBNOCD_00063 hypothetical protein \| DNHOGCFM_00123 hypothetical protein \| Putative ATP-dependent DNA ligase; *Escherichia* phage phAPEC8 \| HLAHOEIE_00155 hypothetical protein \| Putative ATP-dependent DNA ligase; *Escherichia* phage phAPEC8 \| Putative ATP-dependent DNA ligase; *Escherichia* phage phAPEC8 |
| us-ehec-119 | 47771 | 48970 | IFPLOHOB_00109 hypothetical protein \| JOODPJME_00111 hypothetical protein \| JIHLJMCN_00111 hypothetical protein \| OAGBNOCD_00064 hypothetical protein \| HLAHOEIE_00156 hypothetical protein \| hypothetical protein; *Escherichia* phage phAPEC8 |
| us-ehec-120 | 48970 | 49116 | OAGBNOCD_00065 hypothetical protein \| JOODPJME_00110 hypothetical protein \| JIHLJMCN_00112 hypothetical protein \| BDMKCPGI_00112 hypothetical protein \| HFBDACEP_00113 hypothetical protein |
| us-ehec-121 | 49189 | 49380 | OAGBNOCD_00066 hypothetical protein \| JOODPJME_00109 hypothetical protein \| JIHLJMCN_00113 hypothetical protein \| HLAHOEIE_00158 hypothetical protein \| BDMKCPGI_00113 hypothetical protein |
| us-ehec-122 | 49390 | 49716 | OAGBNOCD_00067 hypothetical protein \| JOODPJME_00108 hypothetical protein \| JIHLJMCN_00114 hypothetical protein \| BDMKCPGI_00114 hypothetical protein \| HFBDACEP_00115 hypothetical protein \| Phage protein; ACLAME_Phage_proteins_with_unknown_functions Phage_cyanophage; Phage_experimental; *Enterobacteria* phage phi92 \| Hypothetical protein; Cronobacter phage vb_csam_GAP31 \| AAA domain-containing protein; *Enterobacteria* phage 4MG \| hypothetical protein ECGD1_105; *Enterobacteria* phage ECGD1 |
| us-ehec-123 | 49726 | 50130 | JOODPJME_00107 hypothetical protein \| JIHLJMCN_00115 hypothetical protein \| HLAHOEIE_00160 hypothetical protein \| IFPLOHOB_00113 hypothetical protein \| BDMKCPGI_00115 hypothetical protein \| Phage protein; ACLAME_Phage_proteins_with_unknown_functions Phage_cyanophage; Phage_experimental \| hypothetical protein; *Salmonella* phage SSE-121 \| Phage protein; ACLAME_Phage_proteins_with_unknown_functions Phage_cyanophage; Phage_experimental; *Salmonella* phage PVP-SE1 \| Putative RNA ligase/RNA repair; *Enterobacteria* phage ECGD1 \| Phi92_gp096; *Enterobacteria* phage phi92 \| hypothetical protein; *Salmonella* phage SSE121 \| 106 gene product; *Salmonella* phage PVP-SE1 |
| us-ehec-124 | 50131 | 51063 | OAGBNOCD_00069 hypothetical protein \| JOODPJME_00106 hypothetical protein \| JIHLJMCN_00116 hypothetical protein \| HLAHOEIE_00161 hypothetical protein \| BDMKCPGI_00116 hypothetical protein \| Putative phosphoesterase or phosphohydrolase; *Enterobacteria* phage phi92 \| hypothetical protein; *Escherichia* phage phAPEC8 \| Putative phosphoesterase; *Enterobacteria* phage ECGD1 \| Phi92_gp097; *Enterobacteria* phage phi92 |
| us-ehec-125 | 51065 | 51616 | JOODPJME_00105 hypothetical protein \| JIHLJMCN_00117 hypothetical protein \| HLAHOEIE_00162 hypothetical protein \| BDMKCPGI_00117 hypothetical protein \| HFBDACEP_00118 hypothetical protein \| hypothetical protein; *Escherichia* phage phAPEC8 \| Phage protein; ACLAME_Phage_proteins_with_unknown_functions Phage_cyanophage; Phage_experimental; *Enterobacteria* phage phi92 \| Hypothetical protein phAPEC8_00128; *Escherichia* phage phAPEC8 \| Hypothetical protein ECGD1_108; *Enterobacteria* phage ECGD1 \| Phi92_gp098; *Enterobacteria* phage phi92 |
| us-ehec-126 | 51616 | 51972 | JOODPJME_00104 hypothetical protein \| HLAHOEIE_00163 hypothetical protein \| BDMKCPGI_00118 hypothetical protein \| HFBDACEP_00119 hypothetical protein \| IFPLOHOB_00116 hypothetical protein \| hypothetical protein; *Escherichia* phage phAPEC8 \| Cellulose synthase (EC 2.4.1.12) \| Cellulose synthase (EC 2.4.1.12) \| Cellulose synthase (EC 2.4.1.12) |
| us-ehec-127 | 51979 | 52122 | OAGBNOCD_00071 hypothetical protein \| Hypothetical protein; *Escherichia* phage phAPEC8 \| Hypothetical protein phAPEC8_00130; *Escherichia* phage phAPEC8 |
| us-ehec-128 | 52132 | 52488 | JOODPJME_00102 hypothetical protein \| JIHLJMCN_00120 hypothetical protein \| BDMKCPGI_00120 hypothetical protein \| HFBDACEP_00121 hypothetical protein \| IFPLOHOB_00118 hypothetical protein \| Hypothetical protein; *Escherichia* phage phAPEC8 \| Hypothetical protein phAPEC8_00131; *Escherichia* phage phAPEC8 |
| us-ehec-129 | 52499 | 52969 | HLAHOEIE_00166 hypothetical protein \| JOODPJME_00101 hypothetical protein \| JIHLJMCN_00121 hypothetical protein \| BDMKCPGI_00121 hypothetical protein \| DNHOGCFM_00129 hypothetical protein \| Hypothetical protein; *Escherichia* phage phAPEC8 |
| us-ehec-130 | 52966 | 53172 | HLAHOEIE_00167 hypothetical protein \| DNHOGCFM_00130 hypothetical protein \| hypothetical protein phAPEC8_00133; *Escherichia* phage phAPEC8 \| BDMKCPGI_00122 hypothetical protein \| IFPLOHOB_00120 hypothetical protein \| Hypothetical protein; *Escherichia* phage phAPEC8 |
| us-ehec-131 | 53169 | 53351 | DNHOGCFM_00131 hypothetical protein \| Hypothetical protein phAPEC8_00134; *Escherichia* phage phAPEC8 \| OAGBNOCD_00075 hypothetical protein \| HLAHOEIE_00168 hypothetical protein \| BDMKCPGI_00123 hypothetical protein \| Hypothetical protein; *Escherichia* phage phAPEC8 \| Hypothetical protein ECBP3_0040; *Escherichia* phage KBNP1711 \| Hypothetical protein ECBP2_0039; *Escherichia* phage ECBP2 \| Lipopolysaccharide alpha-1,3-galactosyltransferase (EC  2.4.1.44) |
| us-ehec-132 | 53348 | 53485 | OAGBNOCD_00075 hypothetical protein \| HLAHOEIE_00168 hypothetical protein \| BDMKCPGI_00123 hypothetical protein \| JOODPJME_00099 hypothetical protein \| JIHLJMCN_00123 hypothetical protein \| hypothetical protein; *Escherichia* phage phAPEC8 |
| us-ehec-133 | 53526 | 53630 | IFPLOHOB_00122 hypothetical protein \| JOODPJME_00098 hypothetical protein \| JIHLJMCN_00124 hypothetical protein \| HLAHOEIE_00169 hypothetical protein \| HFBDACEP_00125 hypothetical protein \| Putative transposase-like protein; *Escherichia* phage phAPEC8 \| Putative transposase-like protein ; *Escherichia* phage phAPEC8 |
| us-ehec-134 | 53627 | 53791 | JOODPJME_00097 hypothetical protein \| JIHLJMCN_00125 hypothetical protein \| OAGBNOCD_00077 hypothetical protein \| HLAHOEIE_00170 hypothetical protein \| BDMKCPGI_00126 hypothetical protein \| Hypothetical protein; *Escherichia* phage phAPEC8 |
| us-ehec-135 | 53934 | 54173 | JOODPJME_00096 hypothetical protein \| JIHLJMCN_00126 hypothetical protein \| OAGBNOCD_00078 hypothetical protein \| HLAHOEIE_00171 hypothetical protein \| BDMKCPGI_00127 hypothetical protein \| Hypothetical protein; *Escherichia* phage phAPEC8 |
| us-ehec-136 | 54170 | 54364 | JOODPJME_00095 hypothetical protein \| JIHLJMCN_00127 hypothetical protein \| OAGBNOCD_00079 hypothetical protein \| HLAHOEIE_00172 hypothetical protein \| HFBDACEP_00128 hypothetical protein \| Hypothetical protein ; *Escherichia* phage phAPEC8 |
| us-ehec-137 | 54361 | 54477 | OAGBNOCD_00080 hypothetical protein \| BDMKCPGI_00128 hypothetical protein \| IFPLOHOB_00126 hypothetical protein \| DNHOGCFM_00136 hypothetical protein \| Hypothetical protein phAPEC8_00139; *Escherichia* phage phAPEC8 \| Hypothetical protein; *Escherichia* phage phAPEC8 |
| us-ehec-138 | 54461 | 54784 |  |
| us-ehec-139 | 54884 | 54991 |  |
| us-ehec-140 | 55199 | 55312 | JOODPJME_00093 hypothetical protein \| JIHLJMCN_00129 hypothetical protein \| OAGBNOCD_00081 hypothetical protein \| HLAHOEIE_00174 hypothetical protein \| HFBDACEP_00130 hypothetical protein \| Hypothetical protein; *Escherichia* phage phAPEC8 \| Hypothetical protein; *Cronobacter* phage vb_csam_GAP31 \| Hypothetical protein GAP31_183; *Cronobacter* phage vb_csam_GAP31 |
| us-ehec-141 | 55283 | 55468 | Hypothetical protein phAPEC8_00141; *Escherichia* phage phAPEC8 \| Hypothetical protein; *Escherichia* phage phAPEC8 |
| us-ehec-142 | 55506 | 55757 | JOODPJME_00092 hypothetical protein \| OAGBNOCD_00082 hypothetical protein \| HLAHOEIE_00175 hypothetical protein \| BDMKCPGI_00130 hypothetical protein \| HFBDACEP_00131 hypothetical protein \| Hypothetical protein; *Escherichia* phage phAPEC8 \| T7-like phage ssDNA-binding protein; Pseudomonas phage phi15 \| Putative ssDNA-binding protein; Pseudomonas phage phi15 |
| us-ehec-143 | 55766 | 56014 |  |
| us-ehec-144 | 56087 | 56191 |  |
| us-ehec-145 | 56462 | 56557 |  |
| us-ehec-146 | 56740 | 56889 |  |
| us-ehec-147 | 57143 | 57322 | 4-O-methyl-glucuronoyl methylesterase (EC 3.1.1.-) |
| us-ehec-148 | 57402 | 57509 |  |
| us-ehec-149 | 57649 | 57807 | Hypothetical protein; uncultured Mediterranean UvMED-CGR-U-MedDCM-OCT-S44-C6 |
| us-ehec-150 | 58276 | 58437 | JOODPJME_00081 hypothetical protein \| JIHLJMCN_00141 hypothetical protein \| OAGBNOCD_00094 hypothetical protein \| HLAHOEIE_00186 hypothetical protein \| BDMKCPGI_00141 hypothetical protein \| Hypothetical protein; *Escherichia* phage phAPEC8 \| Hypothetical protein ECGD1_123; *Enterobacteria* phage ECGD1 \| Phi92_gp115; *Enterobacteria* phage phi92 |
| us-ehec-151 | 58595 | 58891 |  |
| us-ehec-152 | 58913 | 59047 | JOODPJME_00080 hypothetical protein \| JIHLJMCN_00142 hypothetical protein \| OAGBNOCD_00095 hypothetical protein \| HLAHOEIE_00187 hypothetical protein \| HFBDACEP_00143 hypothetical protein \| Hypothetical protein; *Escherichia* phage phAPEC8 \| Hypothetical protein phAPEC8_00144; *Escherichia* phage phAPEC8 |
| us-ehec-153 | 59032 | 59202 | JIHLJMCN_00143 hypothetical protein \| BDMKCPGI_00143 hypothetical protein \| Phage protein; ACLAME_Phage_proteins_with_unknown_functions Phage_cyanophage; Phage_experimental; *Enterobacteria* phage phi92 \| Phage tail sheath protein; uncultured Mediterranean phage UvMED-GF-U-MedDCM-OCT-S28-C30 \| Baseplate wedge subunit; *Edwardsiella phage* pei26 \| Baseplate wedge subunit; Edwardsiella *phage* pei20 |
| us-ehec-154 | 59180 | 59269 |  |
| us-ehec-155 | 59293 | 59460 | Hypothetical protein phAPEC8_00146; *Escherichia* phage phAPEC8 \| HFBDACEP_00145 hypothetical protein \| DNHOGCFM_00153 hypothetical protein \| JOODPJME_00078 hypothetical protein \| JIHLJMCN_00145 hypothetical protein \| Hypothetical protein; *Escherichia* phage phAPEC8 |
| us-ehec-156 | 59536 | 59880 | OAGBNOCD_00098 hypothetical protein \| BDMKCPGI_00146 hypothetical protein \| JOODPJME_00077 hypothetical protein \| JIHLJMCN_00146 hypothetical protein \| HLAHOEIE_00190 hypothetical protein \| Putative terminase large subunit; *Escherichia* phage phAPEC8, complete genome \| Putative terminase large subunit; *Escherichia* phage phAPEC8 |
| us-ehec-157 | 59890 | 61965 | JOODPJME_00076 hypothetical protein \| JIHLJMCN_00147 hypothetical protein \| OAGBNOCD_00099 hypothetical protein \| HLAHOEIE_00191 hypothetical protein \| HFBDACEP_00147 hypothetical protein \| Hypothetical protein; *Escherichia* phage phAPEC8 \| Hypothetical protein phAPEC8_00148; *Escherichia* phage phAPEC8] |
| us-ehec-158 | 62067 | 63632 | JOODPJME_00075 hypothetical protein \| JIHLJMCN_00148 hypothetical protein \| OAGBNOCD_00100 hypothetical protein \| HLAHOEIE_00192 hypothetical protein \| BDMKCPGI_00148 hypothetical protein \| Hypothetical protein; *Escherichia* phage phAPEC8 |
| us-ehec-159 | 63726 | 64205 | HLAHOEIE_00193 hypothetical protein \| BDMKCPGI_00149 hypothetical protein \| HFBDACEP_00149 hypothetical protein \| IFPLOHOB_00147 hypothetical protein \| JOODPJME_00074 hypothetical protein \| Hypothetical protein; *Escherichia* phage phAPEC8 \| Phage protein; ACLAME_Phage_proteins_with_unknown_functions Phage_cyanophage; Phage_experimental; *Enterobacteria* phage phi92 |
| us-ehec-160 | 64208 | 64363 | JOODPJME_00074 hypothetical protein \| JIHLJMCN_00149 hypothetical protein \| OAGBNOCD_00101 hypothetical protein \| DNHOGCFM_00157 hypothetical protein \| Hypothetical protein phAPEC8_00150; *Escherichia* phage phAPEC8 \| Hypothetical protein; *Escherichia* phage phAPEC8 |
| us-ehec-161 | 64393 | 65322 | JOODPJME_00073 hypothetical protein \| JIHLJMCN_00150 hypothetical protein \| OAGBNOCD_00102 hypothetical protein \| HLAHOEIE_00194 hypothetical protein \| BDMKCPGI_00150 hypothetical protein \| Putative head stabilization/decoration protein; *Escherichia* phage phAPEC8 \| Putative head stabilization/decoration protein; *Escherichia* phage phAPEC8 |
| us-ehec-162 | 65342 | 65740 | OAGBNOCD_00103 hypothetical protein \| NHOGCFM_00159 hypothetical protein \| Putative major head protein; *Escherichia* phage phAPEC8 \| JOODPJME_00072 hypothetical protein \| JIHLJMCN_00151 hypothetical protein \| Putative major head protein; *Escherichia* phage phAPEC8 \| Elements of external origin phage-related functions and prophages; *Enterobacteria* phage phi92 \| Putative major head protein; *Escherichia* phage phAPEC8 \| Major capsid protein; *Enterobacteria* phage ECGD1 \| Phi92_gp124; *Enterobacteria* phage phi92 |
| us-ehec-163 | 65762 | 66763 | Hypothetical protein phAPEC8_00153; *Escherichia* phage phAPEC8 \| BDMKCPGI_00152 hypothetical protein \| JOODPJME_00071 hypothetical protein \| JIHLJMCN_00152 hypothetical protein \| OAGBNOCD_00104 hypothetical protein \| Hypothetical protein; *Escherichia* phage phAPEC8 |
| us-ehec-164 | 66848 | 67192 | BDMKCPGI_00153 hypothetical protein \| JIHLJMCN_00153 hypothetical protein \| HFBDACEP_00153 hypothetical protein \| IFPLOHOB_00151 hypothetical protein \| JOODPJME_00070 hypothetical protein \| Hypothetical protein; *Escherichia* phage phAPEC8 \| hypothetical protein phAPEC8_00154; *Escherichia* phage phAPEC8 |
| us-ehec-165 | 67202 | 67750 | OAGBNOCD_00106 hypothetical protein \| HFBDACEP_00154 hypothetical protein \| JOODPJME_00069 hypothetical protein \| JIHLJMCN_00154 hypothetical protein \| BDMKCPGI_00154 hypothetical protein \| Hypothetical protein; *Escherichia* phage phAPEC8 \| hypothetical protein phAPEC8_00155; *Escherichia* phage phAPEC8 |
| us-ehec-166 | 67750 | 68238 | JIHLJMCN_00155 hypothetical protein \| OAGBNOCD_00107 hypothetical protein \| HFBDACEP_00155 hypothetical protein \| DNHOGCFM_00163 hypothetical protein \| IFPLOHOB_00153 hypothetical protein \| Hypothetical protein; *Escherichia* phage phAPEC8 \| hypothetical protein phAPEC8_00156; *Escherichia* phage phAPEC8 |
| us-ehec-167 | 68260 | 68700 | JOODPJME_00067 hypothetical protein \| JIHLJMCN_00156 hypothetical protein \| OAGBNOCD_00108 hypothetical protein \| HLAHOEIE_00200 hypothetical protein \| BDMKCPGI_00156 hypothetical protein \| Hypothetical protein; *Escherichia* phage phAPEC8 \| Hypothetical protein phAPEC8_00157; *Escherichia* phage phAPEC8 |
| us-ehec-168 | 68672 | 69331 | JOODPJME_00066 hypothetical protein \| JIHLJMCN_00157 hypothetical protein \| OAGBNOCD_00109 hypothetical protein \| HLAHOEIE_00201 hypothetical protein \| HFBDACEP_00157 hypothetical protein \| Putative structural protein; *Escherichia* phage phAPEC8 \| Putative structural protein; *Escherichia* phage phAPEC8 |
| us-ehec-169 | 69366 | 70739 | JOODPJME_00065 hypothetical protein \| JIHLJMCN_00158 hypothetical protein \| OAGBNOCD_00110 hypothetical protein \| HLAHOEIE_00202 hypothetical protein \| BDMKCPGI_00158 hypothetical protein \| Putative structural protein; *Escherichia* phage phAPEC8 \| Phage protein; ACLAME_Phage_proteins_with_unknown_functions; Phage_cyanophage Phage_experimental; *Enterobacteria* phage phi92 \| Putative structural protein; *Escherichia* phage phAPEC8 \| Putative tail tube; *Enterobacteria* phage ECGD1 \| Phi92_gp131; *Enterobacteria* phage phi92 |
| us-ehec-170 | 70786 | 71265 | JOODPJME_00064 hypothetical protein \| JIHLJMCN_00159 hypothetical protein \| OAGBNOCD_00111 hypothetical protein \| HLAHOEIE_00203 hypothetical protein \| BDMKCPGI_00159 hypothetical protein \| Hypothetical protein; *Escherichia* phage phAPEC8 |
| us-ehec-171 | 71311 | 71793 | Hypothetical protein phAPEC8_00161; *Escherichia* phage phAPEC8 \| Hypothetical protein; *Escherichia* phage phAPEC8 \| Alkaline phosphatase, tissue-nonspecific isozyme; Felis catus \| Phi92_gp133; *Enterobacteria* phage phi92 |
| us-ehec-172 | 71841 | 72059 | JIHLJMCN_00160 hypothetical protein \| HFBDACEP_00160 hypothetical protein \| IFPLOHOB_00158 hypothetical protein \| JOODPJME_00063 hypothetical protein \| DNHOGCFM_00168 hypothetical protein \| hypothetical protein; *Escherichia* phage phAPEC8 |
| us-ehec-173 | 72206 | 74068 | \| |
| us-ehec-174 | 74061 | 74207 | DNHOGCFM_00169 hypothetical protein \| Hypothetical protein phAPEC8_00163; *Escherichia* phage phAPEC8 \| JOODPJME_00062 hypothetical protein \| JIHLJMCN_00161 hypothetical protein \| BDMKCPGI_00161 hypothetical protein \| Hypothetical protein; *Escherichia* phage phAPEC8 \| hypothetical protein phAPEC8_00163; *Escherichia* phage phAPEC8 |
| us-ehec-175 | 74195 | 74896 | JOODPJME_00061 hypothetical protein \| JIHLJMCN_00162 hypothetical protein \| BDMKCPGI_00162 hypothetical protein \| HFBDACEP_00162 hypothetical protein \| DNHOGCFM_00170 hypothetical protein \| Hypothetical protein; *Escherichia* phage phAPEC8 \| hypothetical protein phAPEC8_00164; *Escherichia* phage phAPEC8 |
| us-ehec-176 | 74899 | 75312 | HLAHOEIE_00207 hypothetical protein \| BDMKCPGI_00163 hypothetical protein \| JOODPJME_00060 hypothetical protein \| JIHLJMCN_00163 hypothetical protein \| HFBDACEP_00163 hypothetical protein \| Hypothetical protein; *Escherichia* phage phAPEC8 \| hypothetical protein phAPEC8_00165; *Escherichia* phage phAPEC8 |
| us-ehec-177 | 75322 | 76332 | OAGBNOCD_00116 hypothetical protein \| DNHOGCFM_00172 hypothetical protein \| Hypothetical protein phAPEC8_00166; *Escherichia* phage phAPEC8 \| JIHLJMCN_00164 hypothetical protein \| BDMKCPGI_00164 hypothetical protein \| Hypothetical protein; *Escherichia* phage phAPEC8 \| Hypothetical protein phAPEC8_00166; *Escherichia* phage phAPEC8 |
| us-ehec-178 | 76332 | 77066 | JIHLJMCN_00165 hypothetical protein \| HFBDACEP_00165 hypothetical protein \| IFPLOHOB_00163 hypothetical protein \| JOODPJME_00058 hypothetical protein \| DNHOGCFM_00173 hypothetical protein \| Hypothetical protein; *Escherichia* phage phAPEC8 \| Hypothetical protein phAPEC8_00167; *Escherichia* phage phAPEC8 |
| us-ehec-179 | 77068 | 77694 | JIHLJMCN_00166 hypothetical protein \| BDMKCPGI_00166 hypothetical protein \| HFBDACEP_00166 hypothetical protein \| JOODPJME_00057 hypothetical protein \| OAGBNOCD_00118 hypothetical protein \| Hypothetical protein; *Escherichia* phage phAPEC8 \| Hypothetical protein phAPEC8_00168; *Escherichia* phage phAPEC8 |
| us-ehec-180 | 77694 | 78167 | JIHLJMCN_00167 hypothetical protein \| HFBDACEP_00167 hypothetical protein \| DNHOGCFM_00175 hypothetical protein \| Putative structural protein; *Escherichia* phage phAPEC8 \| IFPLOHOB_00165 hypothetical protein \| Putative structural protein; *Escherichia* phage phAPEC8 \| Putative structural protein; *Escherichia* phage phAPEC8 |
| us-ehec-181 | 78167 | 81043 | BDMKCPGI_00168 hypothetical protein \| OAGBNOCD_00120 hypothetical protein \| JIHLJMCN_00168 hypothetical protein \| HFBDACEP_00168 hypothetical protein \| IFPLOHOB_00166 hypothetical protein \| Hypothetical protein; *Escherichia* phage phAPEC8 |
| us-ehec-182 | 81052 | 83115 | JIHLJMCN_00169 hypothetical protein \| HFBDACEP_00169 hypothetical protein \| IFPLOHOB_00167 hypothetical protein \| DNHOGCFM_00178 hypothetical protein \| Hypothetical protein phAPEC8_00172; *Escherichia* phage phAPEC8 \| Hypothetical protein; *Escherichia* phage phAPEC8 \| Hypothetical protein phAPEC8_00172; *Escherichia* phage phAPEC8 |
| us-ehec-183 | 83108 | 83368 | JOODPJME_00051 hypothetical protein \| JIHLJMCN_00170 hypothetical protein \| HLAHOEIE_00216 hypothetical protein \| IFPLOHOB_00168 hypothetical protein \| HFBDACEP_00170 hypothetical protein \| Hypothetical protein; *Escherichia* phage phAPEC8 \| Hypothetical protein phAPEC8_00173; *Escherichia* phage phAPEC8 |
| us-ehec-184 | 83368 | 84855 | JOODPJME_00050 hypothetical protein \| JIHLJMCN_00171 hypothetical protein \| OAGBNOCD_00127 hypothetical protein \| HLAHOEIE_00217 hypothetical protein \| HFBDACEP_00171 hypothetical protein \| Hypothetical protein; *Escherichia* phage phAPEC8 \| Hypothetical protein phAPEC8_00174; *Escherichia* phage phAPEC8 \| Structural protein; *Enterobacteria* phage ECGD1 |
| us-ehec-185 | 84858 | 85487 | JIHLJMCN_00172 hypothetical protein \| HFBDACEP_00172 hypothetical protein \| IFPLOHOB_00170 hypothetical protein \| DNHOGCFM_00181 hypothetical protein \| Putative gph domain protein; *Escherichia* phage phAPEC8 \| Putative gph domain protein; *Escherichia* phage phAPEC8 |
| us-ehec-186 | 85497 | 86558 | JOODPJME_00048 hypothetical protein \| HFBDACEP_00173 hypothetical protein \| DNHOGCFM_00182 hypothetical protein \| Putative tail fiber assembly protein; *Escherichia* phage phAPEC8 \| JIHLJMCN_00173 hypothetical protein \| Putative tail fiber assembly protein; *Escherichia* phage phAPEC8 \| Putative tail fiber assembly protein ; *Escherichia* phage phAPEC8 |
| us-ehec-187 | 86559 | 87104 | JOODPJME_00047 hypothetical protein \| JIHLJMCN_00174 hypothetical protein \| OAGBNOCD_00130 hypothetical protein \| HLAHOEIE_00220 hypothetical protein \| BDMKCPGI_00175 hypothetical protein \| Hypothetical protein; *Escherichia* phage phAPEC8 |
| us-ehec-188 | 87114 | 87449 | JOODPJME_00046 hypothetical protein \| DNHOGCFM_00184 hypothetical protein \| FPLOHOB_00173 hypothetical protein \| Putative colanic acid-degrading protein; *Escherichia* phage phAPEC8 \| JIHLJMCN_00175 hypothetical protein \| Putative colanic acid-degrading protein; *Escherichia* phage phAPEC8 \| Putative colanic acid-degrading protein; *Escherichia* phage phAPEC8 |
| us-ehec-189 | 87453 | 90506 | DNHOGCFM_00185 hypothetical protein \| Putative phage tail fiber protein; *Escherichia* phage phAPEC8 \| IFPLOHOB_00174 hypothetical protein \| JOODPJME_00045 hypothetical protein \| JIHLJMCN_00176 hypothetical protein \| Putative phage tail fiber protein; *Escherichia* phage phAPEC8 |
| us-ehec-190 | 90568 | 92592 | JIHLJMCN_00177 hypothetical protein \| IFPLOHOB_00175 hypothetical protein \| HFBDACEP_00177 hypothetical protein \| DNHOGCFM_00186 hypothetical protein \| Hypothetical protein phAPEC8_00180; *Escherichia* phage phAPEC8 \| hypothetical protein; *Escherichia* phage phAPEC8 |
| us-ehec-191 | 92634 | 93059 | HFBDACEP_00177 hypothetical protein \| JIHLJMCN_00177 hypothetical protein \| OAGBNOCD_00133 hypothetical protein \| DNHOGCFM_00186 hypothetical protein \| IFPLOHOB_00175 hypothetical protein \| Hypothetical protein; *Escherichia* phage phAPEC8 |
| us-ehec-192 | 93097 | 93426 | \| |
| us-ehec-193 | 93436 | 93588 | HFBDACEP_00177 hypothetical protein \| DNHOGCFM_00186 hypothetical protein \| Hypothetical protein phAPEC8_00180; *Escherichia* phage phAPEC8 \| JOODPJME_00044 hypothetical protein \| IFPLOHOB_00175 hypothetical protein \| hypothetical protein; *Escherichia* phage phAPEC8 |
| us-ehec-194 | 93702 | 95312 | JOODPJME_00043 hypothetical protein \| JIHLJMCN_00179 hypothetical protein \| HFBDACEP_00178 hypothetical protein \| IFPLOHOB_00176 hypothetical protein \| HLAHOEIE_00224 hypothetical protein \| Hypothetical protein; *Escherichia* phage phAPEC8 \| Single-stranded DNA-binding protein 1 ; *Salmonella* typhimurium (strain  LT2 / SGSC1412 / ATCC 700720) \| Single-stranded DNA-binding protein 1; *Salmonella* typhi \| Single-stranded DNA-binding protein; Shigella flexneri \| Hypothetical protein ECGD1_164; *Enterobacteria* phage ECGD1 \| Phi92_gp156; *Enterobacteria* phage phi92 \| Single-stranded DNA-binding protein; *Escherichia* phage RCS47 \| single-stranded DNA-binding protein; Vibrio phage pyd38-A |
| us-ehec-195 | 95376 | 96263 | JOODPJME_00042 hypothetical protein \| JIHLJMCN_00180 hypothetical protein \| HLAHOEIE_00225 hypothetical protein \| HFBDACEP_00179 hypothetical protein \| DNHOGCFM_00188 hypothetical protein \| Hypothetical protein; *Escherichia* phage phAPEC8 |
| us-ehec-196 | 96280 | 96672 | JIHLJMCN_00181 hypothetical protein \| DNHOGCFM_00189 hypothetical protein \| Putative trna nucleotidyl transferase / poly(A) polymerase; *Escherichia* phage  phAPEC8 \| HLAHOEIE_00226 hypothetical protein \| JOODPJME_00041 hypothetical protein \| Putative trna nucleotidyl transferase / poly(A) polymerase; *Escherichia* phage phAPEC8 \| Putative trna nucleotidyl transferase / poly(A) polymerase; *Escherichia* phage phAPEC8 |
| us-ehec-197 | 96718 | 97311 | JIHLJMCN_00182 hypothetical protein \| DNHOGCFM_00190 hypothetical protein \| Hypothetical protein phAPEC8_00184; *Escherichia* phage phAPEC8 \| JOODPJME_00040 hypothetical protein \| OAGBNOCD_00138 hypothetical protein \| Hypothetical protein; *Escherichia* phage phAPEC8 |
| us-ehec-198 | 97304 | 97522 | BDMKCPGI_00183 hypothetical protein \| JIHLJMCN_00183 hypothetical protein \| DNHOGCFM_00191 hypothetical protein \| Hypothetical protein phAPEC8_00185; *Escherichia* phage phAPEC8 \| HLAHOEIE_00228 hypothetical protein \| Hypothetical protein]; *Escherichia* phage phAPEC8 |
| us-ehec-199 | 97533 | 97700 | BDMKCPGI_00184 hypothetical protein \| hypothetical protein; *Escherichia* phage phAPEC8 |
| us-ehec-200 | 97700 | 97891 | JIHLJMCN_00185 hypothetical protein \| HLAHOEIE_00230 hypothetical protein \| DNHOGCFM_00193 hypothetical protein \| Putative DNA polymerase/3'-5' exonuclease domain; *Escherichia* phage phAPEC8 \| IFPLOHOB_00182 hypothetical protein \| Putative DNA polymerase/3'-5' exonuclease domain; *Escherichia* phage phAPEC8 \| Putative DNA polymerase/3'-5' exonuclease domain; *Escherichia* phage phAPEC8 |
| us-ehec-201 | 97941 | 100538 | BDMKCPGI_00186 hypothetical protein \| HFBDACEP_00185 hypothetical protein \| JIHLJMCN_00186 hypothetical protein \| OAGBNOCD_00141 hypothetical protein \| HLAHOEIE_00231 hypothetical protein \| Putative DNA N6-adenine methyltransferase; *Escherichia* phage phAPEC8 \| Putative DNA N6-adenine methyltransferase; *Escherichia* phage phAPEC8 |
| us-ehec-202 | 100538 | 101332 | OAGBNOCD_00142 hypothetical protein \| HLAHOEIE_00232 hypothetical protein \| BDMKCPGI_00187 hypothetical protein \| HFBDACEP_00186 hypothetical protein \| DNHOGCFM_00195 hypothetical protein \| Hypothetical protein; *Escherichia* phage phAPEC8 |
| us-ehec-203 | 101342 | 101773 | JOODPJME_00034 hypothetical protein \| DNHOGCFM_00196 hypothetical protein \| IFPLOHOB_00185 hypothetical protein \| Putative primase/helicase; *Escherichia* phage phAPEC8 \| JIHLJMCN_00188 hypothetical protein \| Putative primase/helicase; *Escherichia* phage phAPEC8 |
| us-ehec-204 | 101866 | 103644 | JOODPJME_00033 hypothetical protein \| JIHLJMCN_00189 hypothetical protein \| OAGBNOCD_00144 hypothetical protein \| HLAHOEIE_00234 hypothetical protein \| BDMKCPGI_00189 hypothetical protein \| Hypothetical protein; *Escherichia* phage phAPEC8 |
| us-ehec-205 | 103657 | 103935 | OAGBNOCD_00145 hypothetical protein \| DNHOGCFM_00198 hypothetical protein \| Hypothetical protein phAPEC8_00192; *Escherichia* phage phAPEC8 \| IFPLOHOB_00187 hypothetical protein \| HLAHOEIE_00235 hypothetical protein \| Hypothetical protein; *Escherichia* phage phAPEC8 \| Sucrose synthase (EC 2.4.1.13) \| Sucrose synthase (EC 2.4.1.13) \| Sucrose synthase (EC 2.4.1.13) \| Sucrose synthase (EC 2.4.1.13) \| Sucrose synthase (EC 2.4.1.13) |
| us-ehec-206 | 103935 | 104879 | OAGBNOCD_00146 hypothetical protein \| JOODPJME_00031 hypothetical protein \| HLAHOEIE_00236 hypothetical protein \| BDMKCPGI_00191 hypothetical protein \| IFPLOHOB_00188 hypothetical protein \| Hypothetical protein; *Escherichia* phage phAPEC8 \| Sucrose synthase (EC 2.4.1.13) \| Sucrose synthase (EC 2.4.1.13) \| Sucrose synthase (EC 2.4.1.13) \| Sucrose synthase (EC 2.4.1.13) \| Sucrose synthase (EC 2.4.1.13) |
| us-ehec-207 | 104887 | 105834 | JIHLJMCN_00192 hypothetical protein \| HFBDACEP_00191 hypothetical protein \| IFPLOHOB_00189 hypothetical protein \| HLAHOEIE_00237 hypothetical protein \| BDMKCPGI_00192 hypothetical protein \| Hypothetical protein; *Escherichia* phage phAPEC8 |
| us-ehec-208 | 105824 | 106507 | JOODPJME_00029 hypothetical protein \| JIHLJMCN_00193 hypothetical protein \| OAGBNOCD_00148 hypothetical protein \| HLAHOEIE_00238 hypothetical protein \| BDMKCPGI_00193 hypothetical protein \| Hypothetical protein; *Escherichia* phage phAPEC8 |
| us-ehec-209 | 106516 | 107079 | JOODPJME_00028 hypothetical protein \| JIHLJMCN_00194 hypothetical protein \| OAGBNOCD_00149 hypothetical protein \| HLAHOEIE_00239 hypothetical protein \| BDMKCPGI_00194 hypothetical protein \| Hypothetical protein; *Escherichia* phage phAPEC8 \| Hypothetical protein phAPEC8_00196; *Escherichia* phage phAPEC8 |
| us-ehec-210 | 107096 | 107287 | JOODPJME_00027 hypothetical protein \| JIHLJMCN_00195 hypothetical protein \| OAGBNOCD_00150 hypothetical protein \| HLAHOEIE_00240 hypothetical protein \| BDMKCPGI_00195 hypothetical protein \| Hypothetical protein; *Escherichia* phage phAPEC8 |
| us-ehec-211 | 107265 | 107543 | JIHLJMCN_00196 hypothetical protein \| OAGBNOCD_00151 hypothetical protein \| HLAHOEIE_00241 hypothetical protein \| HFBDACEP_00195 hypothetical protein \| DNHOGCFM_00204 hypothetical protein \| Hypothetical protein; *Escherichia* phage phAPEC8 \| Hypothetical protein ECGD1_178; *Enterobacteria* phage ECGD1 \| Phi92_gp169; *Enterobacteria* phage phi92 |
| us-ehec-212 | 107495 | 107809 | HLAHOEIE_00242 hypothetical protein \| DNHOGCFM_00205 hypothetical protein \| Putative cell wall hydrolase sleb; *Escherichia* phage phAPEC8 \| OAGBNOCD_00152 hypothetical protein \| JOODPJME_00025 hypothetical protein \| Putative cell wall hydrolase sleb; *Escherichia* phage phAPEC8 \| Putative cell wall hydrolase sleb; *Escherichia* phage phAPEC8 |
| us-ehec-213 | 107781 | 108302 | JOODPJME_00024 General stress protein 16U \| JIHLJMCN_00198 General stress protein 16U \| OAGBNOCD_00153 General stress protein 16U \| HLAHOEIE_00243 General stress protein 16U \| BDMKCPGI_00198 General stress protein 16U \| Hypothetical protein; *Escherichia* phage phAPEC8 \| Hypothetical protein phAPEC8_00200; *Escherichia* phage phAPEC8 |
| us-ehec-214 | 108402 | 109004 | JOODPJME_00023 hypothetical protein \| JIHLJMCN_00199 hypothetical protein \| OAGBNOCD_00154 hypothetical protein \| BDMKCPGI_00199 hypothetical protein \| HFBDACEP_00198 hypothetical protein \| Hypothetical protein; *Escherichia* phage phAPEC8 |
| us-ehec-215 | 109020 | 109289 | OAGBNOCD_00155 hypothetical protein \| HLAHOEIE_00245 hypothetical protein \| HFBDACEP_00199 hypothetical protein \| JOODPJME_00022 hypothetical protein \| JIHLJMCN_00200 hypothetical protein \| Hypothetical protein; *Escherichia* phage phAPEC8 |
| us-ehec-216 | 109270 | 109518 | JOODPJME_00021 hypothetical protein \| JIHLJMCN_00201 hypothetical protein \| HFBDACEP_00200 hypothetical protein \| OAGBNOCD_00156 hypothetical protein \| BDMKCPGI_00201 hypothetical protein \| Hypothetical protein; *Escherichia* phage phAPEC8 \| Hypothetical protein phAPEC8_00203; *Escherichia* phage phAPEC8 |
| us-ehec-217 | 109515 | 110309 | JOODPJME_00020 hypothetical protein \| OAGBNOCD_00157 hypothetical protein \| HLAHOEIE_00247 hypothetical protein \| HFBDACEP_00201 hypothetical protein \| DNHOGCFM_00210 hypothetical protein \| Hypothetical protein; *Escherichia* phage phAPEC8 |
| us-ehec-218 | 110311 | 110589 | DNHOGCFM_00211 hypothetical protein \| Hypothetical protein phAPEC8_00205; *Escherichia* phage phAPEC8 \| OAGBNOCD_00158 hypothetical protein \| JOODPJME_00019 hypothetical protein \| JIHLJMCN_00203 hypothetical protein \| Hypothetical protein; *Escherichia* phage phAPEC8 |
| us-ehec-219 | 110582 | 110893 | HLAHOEIE_00248 hypothetical protein \| DNHOGCFM_00211 hypothetical protein \| Hypothetical protein phAPEC8_00205; *Escherichia* phage phAPEC8 \| JOODPJME_00019 hypothetical protein \| JIHLJMCN_00203 hypothetical protein \| Hypothetical protein; *Escherichia* phage phAPEC8 |
| us-ehec-220 | 110859 | 111146 | DNHOGCFM_00212 hypothetical protein \| Hypothetical protein phAPEC8_00206; *Escherichia* phage phAPEC8 \| IFPLOHOB_00201 hypothetical protein \| JOODPJME_00018 hypothetical protein \| JIHLJMCN_00204 hypothetical protein \| Hypothetical protein; *Escherichia* phage phAPEC8 |
| us-ehec-221 | 111146 | 111544 | JOODPJME_00017 hypothetical protein \| JIHLJMCN_00205 hypothetical protein \| OAGBNOCD_00160 hypothetical protein \| BDMKCPGI_00205 hypothetical protein \| HFBDACEP_00204 hypothetical protein \| Hypothetical protein; *Escherichia* phage phAPEC8 \| hypothetical protein phAPEC8_00207; *Escherichia* phage phAPEC8 |
| us-ehec-222 | 111537 | 111710 | JIHLJMCN_00206 hypothetical protein \| HFBDACEP_00205 hypothetical protein \| DNHOGCFM_00214 hypothetical protein \| Hypothetical protein phAPEC8_00208; *Escherichia* phage phAPEC8 \| HLAHOEIE_00251 hypothetical protein \| Hypothetical protein; *Escherichia* phage phAPEC8 |
| us-ehec-223 | 111710 | 111904 | JOODPJME_00015 tela-like protein \| JIHLJMCN_00207 tela-like protein \| HFBDACEP_00206 tela-like protein \| DNHOGCFM_00215 tela-like protein \| Hypothetical protein phAPEC8_00209; *Escherichia* phage phAPEC8 \| Hypothetical protein; *Escherichia* phage phAPEC8 |
| us-ehec-224 | 111901 | 113013 | HLAHOEIE_00253 hypothetical protein \| IFPLOHOB_00205 hypothetical protein \| Hypothetical protein phAPEC8_00210; *Escherichia* phage phAPEC8 \| JOODPJME_00014 hypothetical protein \| JIHLJMCN_00208 hypothetical protein \| Hypothetical protein; *Escherichia* phage phAPEC8 \| hypothetical protein phAPEC8_00210; *Escherichia* phage phAPEC8 |
| us-ehec-225 | 113010 | 113600 | HLAHOEIE_00254 hypothetical protein \| BDMKCPGI_00209 hypothetical protein \| DNHOGCFM_00217 hypothetical protein \| Hypothetical protein phAPEC8_00211; *Escherichia* phage phAPEC8 \| OAGBNOCD_00164 hypothetical protein \| hypothetical protein; *Escherichia* phage phAPEC8 |
| us-ehec-226 | 113604 | 114197 | Hypothetical protein phAPEC8_00212; *Escherichia* phage phAPEC8 \| DNHOGCFM_00218 hypothetical protein \| OAGBNOCD_00165 hypothetical protein \| JOODPJME_00012 hypothetical protein \| JIHLJMCN_00210 hypothetical protein \| Hypothetical protein; *Escherichia* phage phAPEC8 |
| us-ehec-227 | 114212 | 114724 | HLAHOEIE_00257 hypothetical protein \| JIHLJMCN_00212 hypothetical protein \| HFBDACEP_00211 hypothetical protein \| BDMKCPGI_00212 hypothetical protein \| DNHOGCFM_00220 hypothetical protein \| Hypothetical protein; *Escherichia* phage phAPEC8 |
| us-ehec-228 | 114833 | 115183 | ELONAMNK_00060 Putative membrane-bound redox modulator Alx \| OLDBFMDK_00153 Putative membrane-bound redox modulator Alx \| JHCCGCPF_00030 Putative membrane-bound redox modulator Alx \| OCGFFKNF_00085 Putative membrane-bound redox modulator Alx \| PINPCHNM_00189 Putative membrane-bound redox modulator Alx \| Integral membrane protein terc; *Enterobacteria* phage phi92 \| Phi92_gp182; *Enterobacteria* phage phi92 \| Putative membrane bound tellurium resistance protein; *Enterobacteria* phage ECGD1 |
| us-ehec-229 | 115226 | 116200 | JOODPJME_00008 hypothetical protein \| OAGBNOCD_00169 hypothetical protein \| HLAHOEIE_00259 hypothetical protei \| HFBDACEP_00213 hypothetical protein \| IFPLOHOB_00211 hypothetical protein \| Hypothetical protein; *Escherichia* phage phAPEC8 |
| us-ehec-230 | 116280 | 116501 | DNHOGCFM_00223 hypothetical protein \| Hypothetical protein phAPEC8_00216; *Escherichia* phage phAPEC8 \| JOODPJME_00007 hypothetical protein \| BDMKCPGI_00215 hypothetical protein \| JIHLJMCN_00215 hypothetical protein \| Hypothetical protein; *Escherichia* phage phAPEC8 |
| us-ehec-231 | 116514 | 116987 | OAGBNOCD_00171 hypothetical protein \| DNHOGCFM_00224 hypothetical protein \| Hypothetical protein phAPEC8_00217; *Escherichia* phage phAPEC8 \| JOODPJME_00006 hypothetical protein \| JIHLJMCN_00216 hypothetical protein \| Hypothetical protein; *Escherichia* phage phAPEC8 \| hypothetical protein phAPEC8_00217; *Escherichia* phage phAPEC8 |
| us-ehec-232 | 117001 | 117792 | DNHOGCFM_00225 hypothetical protein \| Hypothetical protein phAPEC8_00218; *Escherichia* phage phAPEC8 \| JOODPJME_00005 hypothetical protein \| HLAHOEIE_00262 hypothetical protein \| Hypothetical protein; *Escherichia* phage phAPEC8 \| Hypothetical protein phAPEC8_00218; *Escherichia* phage phAPEC8 |
| us-ehec-233 | 117822 | 118478 | JOODPJME_00004 hypothetical protein \| OAGBNOCD_00173 hypothetical protein \| HFBDACEP_00217 hypothetical protein \| DNHOGCFM_00226 hypothetical protein \| Hypothetical protein phAPEC8_00219; *Escherichia* phage phAPEC8 \| Hypothetical protein; *Escherichia* phage phAPEC8 |
| us-ehec-234 | 118480 | 119085 | JOODPJME_00003 hypothetical protein \| OAGBNOCD_00174 hypothetical protein \| BDMKCPGI_00219 hypothetical protein \| HFBDACEP_00218 hypothetical protein \| DNHOGCFM_00227 hypothetical protein \| Hypothetical protein; *Escherichia* phage phAPEC8 |
| us-ehec-235 | 119101 | 119292 | DNHOGCFM_00228 Anaerobic ribonucleoside-triphosphate reductase-activating protein \| Ribonucleotide reductase of class III (anaerobic) activating protein; *Escherichia* phage phAPEC8 \| OAGBNOCD_00175 Anaerobic ribonucleoside-triphosphate reductase-activating protein \| JOODPJME_00002 Anaerobic ribonucleoside-triphosphate reductase-activating protein \| BDMKCPGI_00220 Anaerobic ribonucleoside-triphosphate reductase-activating protein \| Ribonucleotide reductase of class III (anaerobic) activating protein |
| us-ehec-236 | 119306 | 119776 | HLAHOEIE_00266 Anaerobic ribonucleoside-triphosphate reductase \| JIHLJMCN_00221 Anaerobic ribonucleoside-triphosphate reductase \| BDMKCPGI_00221 Anaerobic ribonucleoside-triphosphate reductase \| HFBDACEP_00220 Anaerobic ribonucleoside-triphosphate reductase \| OAGBNOCD_00176 Anaerobic ribonucleoside-triphosphate reductase \| Ribonucleotide reductase of class III (anaerobic) large subunit; *Escherichia* phage phAPEC8 \| Ribonucleotide reductase of class III (anaerobic) large subunit; *Escherichia* phage phAPEC8 |
| us-ehec-237 | 119773 | 121587 | HLAHOEIE_00267 hypothetical protein \| JOODPJME_00283 hypothetical protein \| OAGBNOCD_00177 hypothetical protein |
| us-ehec-238 | 121688 | 123034 | JOODPJME_00282 hypothetical protein \| OAGBNOCD_00178 hypothetical protein \| HLAHOEIE_00268 hypothetical protein \| IFPLOHOB_00220 hypothetical protein \| Phage protein; ACLAME_Phage_proteins_with_unknown_functions Phage_cyanophage; Phage_experimental; *Enterobacteria* phage phi92 \| Hypothetical protein; *Escherichia* phage phAPEC8 \| Phi92_gp196; *Enterobacteria* phage phi92 \| Putative seryl-trna synthetase; *Enterobacteria* phage ECGD1 \| Hypothetical protein phAPEC8_00224; *Escherichia* phage phAPEC8 |
| us-ehec-239 | 123031 | 123585 | JOODPJME_00281 hypothetical protein \| JIHLJMCN_00224 hypothetical protein \| HLAHOEIE_00269 hypothetical protein \| BDMKCPGI_00224 hypothetical protein \| HFBDACEP_00223 hypothetical protein \| Hypothetical protein; *Escherichia* phage phAPEC8 \| Hypothetical protein phAPEC8_00225; *Escherichia* phage phAPEC8 |
| us-ehec-240 | 123582 | 123833 | IFPLOHOB_00222 ATP-dependent DNA helicase Rep \| DNHOGCFM_00233 ATP-dependent DNA helicase Rep \| Putative uvrd-type helicase; *Escherichia* phage phAPEC8 \| OAGBNOCD_00180 ATP-dependent DNA helicase Rep \| HLAHOEIE_00270 ATP-dependent DNA helicase Rep \| Putative uvrd-type helicase; *Escherichia* phage phAPEC8 \| Putative uvrd-type helicase; *Escherichia* phage phAPEC8 |
| us-ehec-241 | 123834 | 125426 | JOODPJME_00279 hypothetical protein \| JIHLJMCN_00226 hypothetical protein \| OAGBNOCD_00181 hypothetical protein \| HLAHOEIE_00271 hypothetical protein \| BDMKCPGI_00226 hypothetical protein \| Hypothetical protein; phage phAPEC8 \| hypothetical protein phAPEC8_00227; *Escherichia* phage phAPEC8 |
| us-ehec-242 | 125441 | 125950 | JOODPJME_00278 hypothetical protein \| JIHLJMCN_00227 hypothetical protein \| OAGBNOCD_00182 hypothetical protein \| HLAHOEIE_00272 hypothetical protein \| BDMKCPGI_00227 hypothetical protein \| Hypothetical protein; *Escherichia* phage phAPEC8 \| Hypothetical protein phAPEC8_00228; *Escherichia* phage phAPEC8 |
| us-ehec-243 | 125964 | 126374 | JIHLJMCN_00228 hypothetical protein \| BDMKCPGI_00228 hypothetical protein \| HFBDACEP_00227 hypothetical protein \| HLAHOEIE_00273 hypothetical protein \| JOODPJME_00277 hypothetical protein \| Hypothetical protein; *Escherichia* phage phAPEC8 \| Phage protein; ACLAME_Phage_proteins_with_unknown_functions Phage_cyanophage; Phage_experimental; *Enterobacteria* phage phi92 \| Hypothetical protein phAPEC8_00229; *Escherichia* phage phAPEC8 \| Putative ribonucleoside-diphosphate reductase 1 alpha subunit; *Enterobacteria* phage ECGD1 \| Phi92_gp202; *Enterobacteria* phage phi92 |
| us-ehec-244 | 126314 | 126682 | OAGBNOCD_00184 hypothetical protein \| DNHOGCFM_00237 hypothetical protein \| IFPLOHOB_00226 hypothetical protein \| Hypothetical protein phAPEC8_00230; *Escherichia* phage phAPEC8 \| JOODPJME_00276 hypothetical protein \| Hypothetical protein; *Escherichia* phage phAPEC8 |
| us-ehec-245 | 126684 | 126809 | HLAHOEIE_00274 hypothetical protein \| OAGBNOCD_00184 hypothetical protein \| DNHOGCFM_00237 hypothetical protein \| IFPLOHOB_00226 hypothetical protein \| Hypothetical protein phAPEC8_00230; *Escherichia* phage phAPEC8 \| Hypothetical protein; *Escherichia* phage phAPEC8 |
| us-ehec-246 | 126787 | 127047 | OAGBNOCD_00185 hypothetical protein \| HLAHOEIE_00275 hypothetical protein |
| us-ehec-247 | 127044 | 127406 | OAGBNOCD_00186 hypothetical protein \| HLAHOEIE_00276 hypothetical protein |
| us-ehec-248 | 127407 | 127712 | DNHOGCFM_00239 hypothetical protein \| Hypothetical protein phAPEC8_00232; *Escherichia* phage phAPEC8 \| JOODPJME_00274 hypothetical protein \| JIHLJMCN_00231 hypothetical protein \| BDMKCPGI_00231 hypothetical protein \| Hypothetical protein; *Escherichia* phage phAPEC8 |
| us-ehec-249 | 127712 | 128020 | JOODPJME_00273 hypothetical protein \| JIHLJMCN_00232 hypothetical protein \| HFBDACEP_00231 hypothetical protein \| IFPLOHOB_00229 hypothetical protein \| BDMKCPGI_00232 hypothetical protein \| Hypothetical protein; *Escherichia* phage phAPEC8 \| Hypothetical protein phAPEC8_00233; *Escherichia* phage phAPEC8 |
| us-ehec-250 | 128020 | 128193 | JOODPJME_00272 hypothetical protein \| JIHLJMCN_00233 hypothetical protein \| OAGBNOCD_00189 hypothetical protein \| HLAHOEIE_00279 hypothetical protein \| BDMKCPGI_00233 hypothetical protein \| Hypothetical protein; *Escherichia* phage phAPEC8 \| Terminase; *Listeria* phage LMTA-94 \| Terminase; *Listeria* phage LMTA-57 \| Terminase ; *Listeria* phage LMTA-148 \| gp5; *Listeria* virus P100 \| gp74; *Listeria* virus A511 |
| us-ehec-251 | 128237 | 128434 | HLAHOEIE_00280 hypothetical protein \| DNHOGCFM_00242 hypothetical protein \| Hypothetical protein phAPEC8_00235; *Escherichia* phage phAPEC8 \| JOODPJME_00271 hypothetical protein \| JIHLJMCN_00234 hypothetical protein \| Hypothetical protein; *Escherichia* phage phAPEC8 |
| us-ehec-252 | 128517 | 128786 | JOODPJME_00270 hypothetical protein \| JIHLJMCN_00235 hypothetical protein \| OAGBNOCD_00191 hypothetical protein \| BDMKCPGI_00235 hypothetical protein \| HFBDACEP_00234 hypothetical protein \| Hypothetical protein; *Escherichia* phage phAPEC8 |
| us-ehec-253 | 128783 | 128980 |  |
| us-ehec-254 | 128967 | 129062 | JOODPJME_00269 hypothetical protein \| JIHLJMCN_00236 hypothetical protein \| BDMKCPGI_00236 hypothetical protein \| HFBDACEP_00235 hypothetical protein \| IFPLOHOB_00233 hypothetical protein \| Hypothetical protein; *Escherichia* phage phAPEC8 |
| us-ehec-255 | 129150 | 129473 | JOODPJME_00268 hypothetical protein \| JIHLJMCN_00237 hypothetical protein \| HLAHOEIE_00283 hypothetical protein \| BDMKCPGI_00237 hypothetical protein \| HFBDACEP_00236 hypothetical protein \| Hypothetical protein; *Escherichia* phage phAPEC8 \| Hypothetical protein phAPEC8_00238 ; *Escherichia* phage phAPEC8 |
| us-ehec-256 | 129466 | 129615 | HLAHOEIE_00284 hypothetical protein \| OAGBNOCD_00194 hypothetical protein \| DNHOGCFM_00246 hypothetical protein \| Hypothetical protein phAPEC8_00239; *Escherichia* phage phAPEC8 \| Hypothetical protein; *Escherichia* phage phAPEC8 |
| us-ehec-257 | 129675 | 129935 | DNHOGCFM_00247 hypothetical protein \| Hypothetical protein phAPEC8_00240; *Escherichia* phage phAPEC8 \| ELONAMNK_00032 hypothetical protein \| OLDBFMDK_00125 hypothetical protein \| JHCCGCPF_00058 hypothetical protein \| Hypothetical protein; *Escherichia* phage phAPEC8 \| Phage protein; ACLAME_Phage_proteins_with_unknown_functions Phage_cyanophage; Phage_experimental; *Enterobacteria* phage phi92 \| Hypothetical protein phAPEC8_00240; *Escherichia* phage phAPEC8 \| Phi92_gp213; *Enterobacteria* phage phi92 \| Hypothetical protein ECGD1_220; *Enterobacteria* phage ECGD1 |
| us-ehec-258 | 129932 | 130084 |  |
| us-ehec-259 | 130087 | 130710 |  |
| us-ehec-260 | 130632 | 131051 | JOODPJME_00264 hypothetical protein \| JIHLJMCN_00241 hypothetical protein \| HFBDACEP_00240 hypothetical protein \| IFPLOHOB_00238 hypothetical protein \| OAGBNOCD_00196 hypothetical protein \| Hypothetical protein; *Escherichia* phage phAPEC8 \| Hyphothetical protein; *Escherichia* phage 4MG |
| us-ehec-261 | 131103 | 131528 |  |
| us-ehec-262 | 131529 | 131630 | JOODPJME_00263 hypothetical protein \| JIHLJMCN_00242 hypothetical protein \| OAGBNOCD_00197 hypothetical protein \| BDMKCPGI_00242 hypothetical protein \| HFBDACEP_00241 hypothetical protein \| Hypothetical protein; *Escherichia* phage phAPEC8 \| Hypothetical protein phAPEC8_00242; *Escherichia* phage phAPEC8 |
| us-ehec-263 | 131646 | 131936 | DNHOGCFM_00250 hypothetical protein \| Hypothetical protein phAPEC8_00243; *Escherichia* phage phAPEC8 \| Hypothetical protein; *Escherichia* phage phAPEC8 \| Hypothetical protein phAPEC8_00243; *Escherichia* phage phAPEC8 |
| us-ehec-264 | 131949 | 132440 | DNHOGCFM_00251 hypothetical protein \| Hypothetical protein phAPEC8_0024; *Escherichia* phage phAPEC8 \| OAGBNOCD_00198 hypothetical protein \| HLAHOEIE_00002 hypothetical protein \| BDMKCPGI_00243 hypothetical protein \| Hypothetical protein; *Escherichia* phage phAPEC8 \| Phage protein; ACLAME_Phage_proteins_with_unknown_functions Phage_cyanophage; Phage_experimental; *Enterobacteria* phage phi92 \| Hypothetical protein phAPEC8_00244; *Escherichia* phage phAPEC8 |
| us-ehec-265 | 132478 | 132765 | JOODPJME_00261 hypothetical protein \| JIHLJMCN_00244 hypothetical protein \| HLAHOEIE_00003 hypothetical protein \| BDMKCPGI_00244 hypothetical protein \| HFBDACEP_00243 hypothetical protein \| Hypothetical protein; *Escherichia* phage phAPEC8 |
| us-ehec-266 | 132762 | 133118 | AGBNOCD_00200 hypothetical protein \| BDMKCPGI_00245 hypothetical protein \| IFPLOHOB_00242 hypothetical protein \| JOODPJME_00260 hypothetical protein \| JIHLJMCN_00245 hypothetical protein \| Hypothetical protein; *Escherichia* phage phAPEC8 |
| us-ehec-267 | 133121 | 133297 | JOODPJME_00259 hypothetical protein \| JIHLJMCN_00246 hypothetical protein \| OAGBNOCD_00201 hypothetical protein \| HLAHOEIE_00005 hypothetical protein \| BDMKCPGI_00246 hypothetical protein \| Hypothetical protein; *Escherichia* phage phAPEC8 \| Hypothetical protein phAPEC8_00247; *Escherichia* phage phAPEC8 \| Hypothetical protein ECGD1_225; *Enterobacteria* phage ECGD1 |
| us-ehec-268 | 133308 | 133646 | OAGBNOCD_00202 hypothetical protein \| HLAHOEIE_00006 hypothetical protein \| DNHOGCFM_00255 hypothetical protein \| Hypothetical protein phAPEC8_00248; *Escherichia* phage phAPEC8 \| JOODPJME_00258 hypothetical protein \| Hypothetical protein; *Escherichia* phage phAPEC8 |
| us-ehec-269 | 133646 | 133897 | JOODPJME_00257 hypothetical protein \| JIHLJMCN_00248 hypothetical protein \| OAGBNOCD_00203 hypothetical protein \| BDMKCPGI_00248 hypothetical protein \| HFBDACEP_00247 hypothetical protein \| Hypothetical protein; *Escherichia* phage phAPEC8 |
| us-ehec-270 | 133911 | 134075 | JOODPJME_00256 hypothetical protein \| JIHLJMCN_00249 hypothetical protein \| OAGBNOCD_00204 hypothetical protein \| BDMKCPGI_00249 hypothetical protein \| HFBDACEP_00248 hypothetical protein \| Hypothetical protein; *Escherichia* phage phAPEC8 |
| us-ehec-271 | 134085 | 134249 | BDMKCPGI_00250 hypothetical protein \| OAGBNOCD_00205 hypothetical protein \| HLAHOEIE_00009 hypothetical protein \| HFBDACEP_00249 hypothetical protein \| IFPLOHOB_00247 hypothetical protein \| Hypothetical protein; *Escherichia* phage phAPEC8 |
| us-ehec-272 | 134261 | 134617 | JOODPJME_00254 hypothetical protein \| JIHLJMCN_00251 hypothetical protein \| OAGBNOCD_00206 hypothetical protein \| HLAHOEIE_00010 hypothetical protein \| BDMKCPGI_00251 hypothetical protein \| Putative atpase; *Escherichia* phage phAPEC8 \| Putative atpase; *Escherichia* phage phAPEC8 |
| us-ehec-273 | 134617 | 135081 | IFPLOHOB_00248 hypothetical protein \| JOODPJME_00254 hypothetical protein \| JIHLJMCN_00251 hypothetical protein \| OAGBNOCD_00206 hypothetical protein \| HLAHOEIE_00010 hypothetical protein \| Putative atpase; *Escherichia* phage phAPEC8 \| Putative atpase; *Escherichia* phage phAPEC8 |
| us-ehec-274 | 135219 | 135758 | DNHOGCFM_00260 hypothetical protein \| Hypothetical protein phAPEC8_00253; *Escherichia* phage phAPEC8 \| JOODPJME_00253 hypothetical protein \| HLAHOEIE_00011 hypothetical protein \| Hypothetical protein; *Escherichia* phage phAPEC8 \| Hypothetical protein phAPEC8_00253; *Escherichia* phage phAPEC8 |
| us-ehec-275 | 135837 | 136835 | JOODPJME_00252 hypothetical protein \| JIHLJMCN_00253 hypothetical protein \| OAGBNOCD_00208 hypothetical protein \| HLAHOEIE_00012 hypothetical protein \| BDMKCPGI_00253 hypothetical protein \| Hypothetical protein; *Escherichia* phage phAPEC8 |
| us-ehec-276 | 136829 | 137194 | DNHOGCFM_00262 hypothetical protein \| Hypothetical protein phAPEC8_00255; *Escherichia* phage phAPEC8 \| OAGBNOCD_00209 hypothetical protein \| JOODPJME_00251 hypothetical protein \| JIHLJMCN_00254 hypothetical protein \| Hypothetical protein; *Escherichia* phage phAPEC8 |
| us-ehec-277 | 137208 | 137396 | OAGBNOCD_00210 hypothetical protein \| BDMKCPGI_00255 hypothetical protein \| DNHOGCFM_00263 hypothetical protein \| Hypothetical protein phAPEC8_00256; *Escherichia* phage phAPEC8 \| HLAHOEIE_00014 hypothetical protein \| Hypothetical protein; *Escherichia* phage phAPEC8 \| Hypothetical protein phAPEC8_00256; *Escherichia* phage phAPEC8 |
| us-ehec-278 | 137383 | 137925 |  |
| us-ehec-279 | 137957 | 138094 |  |
| us-ehec-280 | 138224 | 138370 |  |
| us-ehec-281 | 138442 | 138561 |  |
| us-ehec-282 | 138619 | 138762 | SNF2 family helicase; *Bacillus* phage SP-10 \| |
| us-ehec-283 | 138763 | 138858 |  |
| us-ehec-284 | 138906 | 139139 |  |
| us-ehec-285 | 139136 | 139252 | OAGBNOCD_00211 hypothetical protein \| JOODPJME_00249 hypothetical protein \| JIHLJMCN_00256 hypothetical protein \| BDMKCPGI_00257 hypothetical protein \| IFPLOHOB_00253 hypothetical protein \| Hypothetical protein; *Escherichia* phage phAPEC8 |
| us-ehec-286 | 139270 | 139467 | BDMKCPGI_00258 hypothetical protein \| Hypothetical protein phAPEC8_00259; *Escherichia* phage phAPEC8 \| DNHOGCFM_00266 hypothetical protein \| JIHLJMCN_00257 hypothetical protein \| JOODPJME_00248 hypothetical protein \| Hypothetical protein; *Escherichia* phage phAPEC8 \| Phage protein; ACLAME_Phage_proteins_with_unknown_functions Phage_cyanophage; Phage_experimental \| Hypothetical protein phAPEC8_00259; *Escherichia* phage phAPEC8 \| Phi92_gp226; *Enterobacteria* phage phi92 \| Hypothetical protein ECGD1_001; *Enterobacteria* phage ECGD1 |
| us-ehec-287 | 139559 | 139831 | JOODPJME_00247 hypothetical protein \| JIHLJMCN_00258 hypothetical protein \| OAGBNOCD_00213 hypothetical protein \| BDMKCPGI_00259 hypothetical protein \| DNHOGCFM_00267 hypothetical protein \| Hypothetical protein; *Escherichia* phage phAPEC8 |
| us-ehec-288 | 139908 | 140165 | IFPLOHOB_00256 hypothetical protein \| DNHOGCFM_00268 hypothetical protein \| hypothetical protein phAPEC8_00261; *Escherichia* phage phAPEC8 \| JOODPJME_00246 hypothetical protein \| JIHLJMCN_00259 hypothetical protein \| Hypothetical protein; *Escherichia* phage phAPEC8 |
| us-ehec-289 | 140167 | 140724 | HLAHOEIE_00020 hypothetical protein \| JIHLJMCN_00260 hypothetical protein \| JOODPJME_00245 hypothetical protein \| HFBDACEP_00259 hypothetical protein \| IFPLOHOB_00257 hypothetical protein |
| us-ehec-290 | 140768 | 140902 | JIHLJMCN_00261 hypothetical protein \| JLLHFBGM_00240 hypothetical protein \| OAGBNOCD_00215 hypothetical protein \| BDMKCPGI_00261 hypothetical protein \| IFPLOHOB_00258 hypothetical protein \| Hypothetical protein; *Escherichia* phage 121Q \| hypothetical protein; *Escherichia* phage 2 JES-2013 \| Phage protein; ACLAME_Phage_proteins_with_unknown_functions Phage_cyanophage; Phage_experimental; *Enterobacteria* phage vb_ecom-FV3 \| Hypothetical protein; *Escherichia* phage vb_ecom_FFH2 \| Phage protein; ACLAME_Phage_proteins_with_unknown_functions; Phage_cyanophage Phage_experimental; Myoviridae *Escherichia* phage rv5 \| Phi92_gp227; *Enterobacteria* phage phi92 \| Hypothetical protein PBI_121Q_266; *Escherichia* phage 121Q \| Hypothetical protein; *Escherichia* phage slur12 \| Hypothetical protein Ec2_00173; *Escherichia* phage JES2013 \| Hypothetical protein; *Escherichia* phage 2 JES-2013 |
| us-ehec-291 | 140994 | 141308 | JOODPJME_00243 hypothetical protein \| JIHLJMCN_00262 hypothetical protein \| HFBDACEP_00261 hypothetical protein \| IFPLOHOB_00259 hypothetical protein \| Hypothetical protein; *Escherichia* phage vb_ecom_FFH2 \| Hypothetical protein; *Escherichia* phage 2 JES-2013 \| Phage protein; ACLAME_Phage_proteins_with_unknown_functions Phage_cyanophage Phage_experimental; Myoviridae *Escherichia* phage rv5 \| Hypothetical protein CPT_Murica160; *Escherichia* phage Murica \| Hypothetical protein; *Escherichia* phage V5 \| Hypothetical protein ECTP5_00861; *Escherichia* coli O157 typing phage 5 \| Hypothetical protein; *Escherichia* phage slur12 \| Hypothetical protein Ec2_00165; *Escherichia* phage JES2013 |
| us-ehec-292 | 141441 | 141620 | JOODPJME_00242 hypothetical protein \| JIHLJMCN_00263 hypothetical protein \| HFBDACEP_00262 hypothetical protein \| BDMKCPGI_00262 hypothetical protein \| IFPLOHOB_00260 hypothetical protein \| Phage protein; ACLAME_Phage_proteins_with_unknown_functions Phage_cyanophage; Phage_experimental ; *Enterobacteria* phage phi92 \| Hypothetical protein ECGD1_005; *Enterobacteria* phage ECGD1 \| Phi92_gp231; *Enterobacteria* phage phi92 |
| us-ehec-293 | 141718 | 142083 | JOODPJME_00241 hypothetical protein \| JIHLJMCN_00264 hypothetical protein \| HFBDACEP_00263 hypothetical protein \| JEGPDCFI_00304 hypothetical protein \| Hypothetical protein; *Escherichia* phage PBECO 4 \| hypothetical protein; *Escherichia* phage PBECO 4 \| hypothetical protein; *Escherichia* phage 121Q \| Phage protein; ACLAME_Phage_proteins_with_unknown_functions Phage_cyanophage; Phage_experimental; *Enterobacteria* phage phi92 \| Hypothetical protein; *Escherichia* phage PBECO 4 \| Hypothetical protein PBI_121Q_267; *Escherichia* phage 121Q \| Phi92_gp232; *Enterobacteria* phage phi92 |
| us-ehec-294 | 142173 | 142412 | HLAHOEIE_00024 hypothetical protein \| IFPLOHOB_00262 hypothetical protein \| JOODPJME_00240 hypothetical protein \| JIHLJMCN_00265 hypothetical protein \| OAGBNOCD_00218 hypothetical protein \| Hypothetical protein; *Escherichia* phage phAPEC8 \| Hypothetical protein phAPEC8_00264; *Escherichia* phage phAPEC8 \| Phi92_gp233; *Enterobacteria* phage phi92 |
| us-ehec-295 | 142504 | 142731 | LNGMFLFM_00034 hypothetical protein \| CBHAPDHH_00034 hypothetical protein \| Tail protein; Arthrobacter phage kellezio \| Tail protein; *Arthrobacter* phage Kitkat |
| us-ehec-296 | 142790 | 142879 |  |
| us-ehec-297 | 143054 | 143239 | IFPLOHOB_00266 hypothetical protein \| OAGBNOCD_00221 hypothetical protein \| HFBDACEP_00267 hypothetical protein \| DNHOGCFM_00274 hypothetical protein \| Hypothetical protein phAPEC8_00267; *Escherichia* phage phAPEC8 \| Hypothetical protein; *Escherichia* phage phAPEC8 |
| us-ehec-298 | 143375 | 143596 | JOODPJME_00235 hypothetical protein \| JIHLJMCN_00270 hypothetical protein \| OAGBNOCD_00222 hypothetical protein \| HLAHOEIE_00028 hypothetical protein \| HFBDACEP_00268 hypothetical protein \| Hypothetical protein; *Escherichia* phage phAPEC8 \| Hypothetical protein phAPEC8_00268; *Escherichia* phage phAPEC8 |
| us-ehec-299 | 143676 | 143921 |  |
| us-ehec-300 | 143937 | 144143 |  |
| us-ehec-301 | 144121 | 144276 | OAGBNOCD_00224 hypothetical protein \| HFBDACEP_00270 hypothetical protein \| JOODPJME_00233 hypothetical protein \| JIHLJMCN_00272 hypothetical protein \| IFPLOHOB_00269 hypothetical protein \| Hypothetical protein; *Escherichia* phage phAPEC8 \| ATG4C_XENTR Cysteine protease; *Xenopus tropicalis* |
| us-ehec-302 | 144313 | 144498 | OAGBNOCD_00225 hypothetical protein \| HLAHOEIE_00031 hypothetical protein \| Hypothetical protein; *Escherichia* phage phAPEC8 |
| us-ehec-303 | 144584 | 144790 | DNHOGCFM_00003 hypothetical protein \| HFBDACEP_00272 hypothetical protein \| JOODPJME_00231 hypothetical protein \| IFPLOHOB_00271 hypothetical protein \| JIHLJMCN_00274 hypothetical protein \| Hypothetical protein; *Escherichia* phage phAPEC8 |
| us-ehec-304 | 144832 | 145146 | HLAHOEIE_00033 hypothetical protein \| HFBDACEP_00273 hypothetical protein \| IFPLOHOB_00272 hypothetical protein \| DNHOGCFM_00004 hypothetical protein \| Hypothetical protein phAPEC8_004; *Escherichia* phage phAPEC8 \| Hypothetical protein; *Escherichia* phage phAPEC8 \| Hypothetical protein phAPEC8_004; *Escherichia* phage phAPEC8 |
| us-ehec-305 | 145244 | 145363 | JIHLJMCN_00276 hypothetical protein \| OAGBNOCD_00227 hypothetical protein \| HLAHOEIE_00034 hypothetical protein \| HFBDACEP_00274 hypothetical protein \| IFPLOHOB_00273 hypothetical protein \| Hypothetical protein; *Escherichia* phage phAPEC8 |
| us-ehec-306 | 145374 | 145535 | OAGBNOCD_00228 hypothetical protein \| Hypothetical protein phAPEC8_007; *Escherichia* phage phAPEC8 \| Hypothetical protein; *Escherichia* phage phAPEC8 |
| us-ehec-307 | 145545 | 145655 | JOODPJME_00229 hypothetical protein \| JIHLJMCN_00277 hypothetical protein \| DCKEIKPP_00012 hypothetical protein \| JLLHFBGM_00253 hypothetical protein \| OAGBNOCD_00229 hypothetical protein \| Hypothetical protein; *Escherichia* phage phAPEC8 \| Phage protein; ACLAME_Phage_proteins_with_unknown_functions Phage_cyanophage; Phage_experimental; *Enterobacteria* phage phi92 \| Hypothetical protein phAPEC8_008; *Escherichia* phage phAPEC8 \| Phi92_gp242; *Enterobacteria* phage phi92 |
| us-ehec-308 | 145683 | 146018 | DNHOGCFM_00008 hypothetical protein \| JOODPJME_00228 hypothetical protein \| ELONAMNK_00004 hypothetical protein \| HLAHOEIE_00036 hypothetical protein \| OLDBFMDK_00097 hypothetical protein \| Hypothetical protein; *Escherichia* phage phAPEC8 |
| us-ehec-309 | 146104 | 146274 |  |
| us-ehec-310 | 146264 | 146380 | DNHOGCFM_00009 hypothetical protein \| NNKEDCCG_00006 hypothetical protein \| PKGENPCH_00138 hypothetical protein \| MILOOAOH_00213 hypothetical protein \| NLAEJCGN_00007 hypothetical protein \| Phage protein; ACLAME_Phage_proteins_with_unknown_functions Phage_cyanophage; Phage_experimental; *Enterobacteria* phage vb_ecom-FV3 |
| us-ehec-311 | 146441 | 146644 | JIHLJMCN_00280 hypothetical protein \| HLAHOEIE_00038 hypothetical protein \| HFBDACEP_00278 hypothetical protein \| DNHOGCFM_00010 hypothetical protein \| IFPLOHOB_00278 hypothetical protein \| Hypothetical protein; *Escherichia* phage phAPEC8 \| Hypothetical protein phAPEC8_0010; *Escherichia* phage phAPEC8 \| Hypothetical protein apcec02_060; *Escherichia* phage apcec02 |
| us-ehec-312 | 146655 | 146870 | BDMKCPGI_00267 hypothetical protein \| DNHOGCFM_00011 hypothetical protein \| Hypothetical protein phAPEC8_0011; *Escherichia* phage phAPEC8 \| HLAHOEIE_00039 hypothetical protein \| DCKEIKPP_00008 hypothetical protein \| Hypothetical protein; *Escherichia* phage phAPEC8 \| Hypothetical protein phAPEC8_0011; *Escherichia* phage phAPEC8 |
| us-ehec-313 | 146867 | 147253 | HLAHOEIE_00040 hypothetical protein \| IFPLOHOB_00280 hypothetical protein \| OAGBNOCD_00234 hypothetical protein |
| us-ehec-314 | 147337 | 147528 | JOODPJME_00224 hypothetical protein \| JIHLJMCN_00282 hypothetical protein \| DCKEIKPP_00007 hypothetical protein \| HLAHOEIE_00041 hypothetical protein \| BDMKCPGI_00268 hypothetical protein \| Hypothetical protein; *Escherichia* phage phAPEC8 |
| us-ehec-315 | 147607 | 147906 | PINPCHNM_00249 hypothetical protein \| Phi92_gp244; *Enterobacteria* phage phi92 \| DNHOGCFM_00013 hypothetical protein \| Hypothetical protein phAPEC8_0013; *Escherichia* phage phAPEC8 \| JOODPJME_00223 hypothetical protein \| Phage protein; ACLAME_Phage_proteins_with_unknown_functions Phage_cyanophage; Phage_experimental; *Enterobacteria* phage phi92 \| Hypothetical protein; *Escherichia* phage phAPEC8 |
| us-ehec-316 | 147951 | 148187 | DCKEIKPP_00005 hypothetical protein \| DNHOGCFM_00014 hypothetical protein \| Hypothetical protein phAPEC8_0014; *Escherichia* phage phAPEC8 \| HLAHOEIE_00042 hypothetical protein \| OAGBNOCD_00236 hypothetical protein \| Hypothetical protein; *Escherichia* phage phAPEC8 \| Hypothetical protein; *Escherichia* phage vb_ecom_FFH2 |
| us-ehec-317 | 148243 | 148527 | HLAHOEIE_00043 hypothetical protein \| OAGBNOCD_00237 hypothetical protein \| Hypothetical protein; *Escherichia* phage 2 JES-2013 \| Hypothetical protein; *Escherichia* phage phAPEC8 |
| us-ehec-318 | 148530 | 148664 | Phage terminase, small subunit; ACLAME_Phage_head Phage_packaging_machinery T4-like_phage_core_proteins zzrobe_test; Caudovirales *Geobacillus* phage GBSV1 \| Phage terminase, small subunit; ACLAME_Phage_head Phage_packaging_machinery T4-like_phage_core_proteins zzrobe_test; Caudovirales *Bacillus* virus 1 \| Hypothetical protein BV1_gp17; *Bacillus* virus 1 \| Hypothetical protein GPGV1_gp16; *Geobacillus* phage GBSV1 \| beta-galactosidase (EC 3.2.1.23) |
| us-ehec-319 | 148681 | 148779 | HFBDACEP_00283 hypothetical protein \| JIHLJMCN_00285 hypothetical protein \| HLAHOEIE_00044 hypothetical protein \| BDMKCPGI_00271 hypothetical protein \| PEGFCKOL_00190 hypothetical protein \| Hypothetical protein; *Escherichia* phage 121Q \| Hypothetical protein PBI_121Q_272; *Escherichia* phage 121Q |
| us-ehec-320 | 148917 | 149027 |  |
| us-ehec-321 | 149051 | 149173 | JIHLJMCN_00286 hypothetical protein \| NHOGCFM_00015 hypothetical protein \| hypothetical protein phAPEC8_0016; *Escherichia* phage phAPEC8 \| HLAHOEIE_00045 hypothetical protein \| HFBDACEP_00284 hypothetical protein \| Hypothetical protein; *Escherichia* phage phAPEC8 |
| us-ehec-322 | 149230 | 149598 | Hypothetical protein; *Bacillus* phage Spock \| Hypothetical protein Spock_150; *Bacillus* phage Spock |
| us-ehec-323 | 149650 | 149742 | HLAHOEIE_00046 hypothetical protein \| DNHOGCFM_00016 hypothetical protein \| Hypothetical protein phAPEC8_0017; *Escherichia* phage phAPEC8 \| JOODPJME_00218 hypothetical protein \| JIHLJMCN_00287 hypothetical protein \| Hypothetical protein; *Escherichia* phage phAPEC8 \| Phage protein; ACLAME_Phage_proteins_with_unknown_functions Phage_cyanophage; Phage_experimental; Myoviridae *Escherichia* phage rv5 \| Hypothetical protein; *Escherichia* phage vb_ecom_FFH2 \| Hypothetical protein; *Escherichia* phage 2 JES-2013 |
| us-ehec-324 | 149739 | 150158 |  |
| us-ehec-325 | 150159 | 150260 |  |
| us-ehec-326 | 150286 | 150384 |  |
| us-ehec-327 | 150460 | 150570 |  |
| us-ehec-328 | 150571 | 150780 |  |
| us-ehec-329 | 150855 | 151025 |  |
| us-ehec-330 | 151159 | 151248 |  |
| us-ehec-331 | 151303 | 151413 |  |
| us-ehec-332 | 56261 | 56335 | tRNA-Ile2(CAT) |
| us-ehec-333 | 56338 | 56412 | tRNA-Arg(TCT) |
| us-ehec-334 | 56647 | 56737 | tRNA-Ser(TGA) |
| us-ehec-335 | 56743 | 56828 | tRNA-Ser(GCT) |
| us-ehec-336 | 56960 | 57047 | tRNA-Tyr(GTA) |
| us-ehec-337 | 57054 | 57139 | tRNA-Asn(GTT) |
| us-ehec-338 | 57341 | 57416 | tRNA-Thr(TGT) |
| us-ehec-339 | 57733 | 57806 | tRNA-Gly(TCC) |
| us-ehec-340 | 57905 | 57980 | tRNA-Gln(TTG) |
| us-ehec-341 | 58074 | 58150 | tRNA-Pro(TGG) |
| us-ehec-342 | 58157 | 58230 | tRNA-Ile(GAT) |
| us-ehec-343 | 58309 | 58387 | tRNA-fmet(CAT) |
