# Supplementary material for: Genomic characterization of bacteriophage BI-EHEC infecting strains of Enterohemorrhagic Escherichia coli
Source: BMC Res Notes. 2021 Dec 20;14:459. doi: 10.1186/s13104-021-05881-5 (PMC8686590; doi:10.1186/s13104-021-05881-5)
Supplement: Supplementary file 2 — Additional file 2. Figure S1 Genome map of BI-EHEC. Annotation was selected based on its role on lyric cycle and/or structural. [file 13104_2021_5881_MOESM2_ESM.docx]

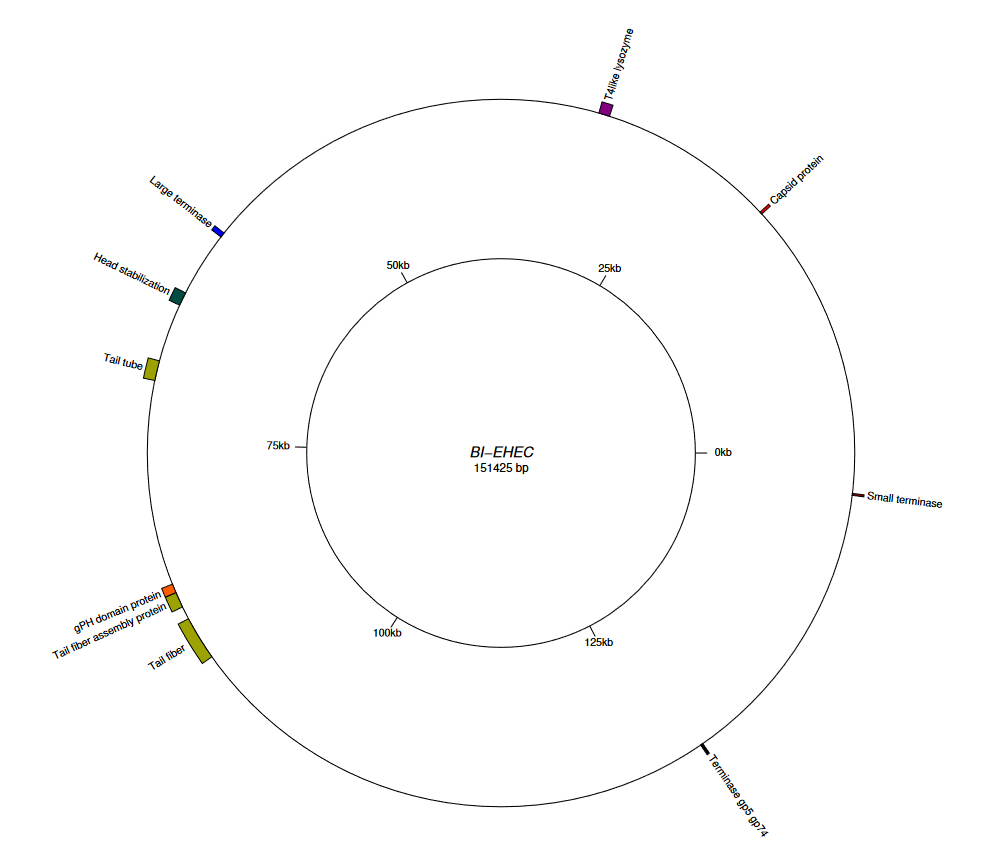


Figure S1 Genome map of BI-EHEC. Annotation was selected based on its role on lyric cycle and/or structural.
