# Supplementary material for: Genomic characterization of bacteriophage BI-EHEC infecting strains of Enterohemorrhagic Escherichia coli
Source: BMC Res Notes. 2021 Dec 20;14:459. doi: 10.1186/s13104-021-05881-5 (PMC8686590; doi:10.1186/s13104-021-05881-5)
Supplement: Supplementary file 3 — Additional file 3. Table S2 List of tail fiber from NCBI database and its accession number. [file 13104_2021_5881_MOESM3_ESM.docx]

Table S2 List of tail fiber from NCBI database and its accession number

| Phage name | Host | NCBI Accession number |
| --- | --- | --- |
| *Escherichia* phage muut | *Escherichia* *coli* K-12 MG1655 | NC_052657.1 |
| *Escherichia* phage VEcB | *Escherichia* *coli* | NC_052663.1 |
| *Escherichia* phage vB_vPM_PD114 | *Escherichia* *coli* | MH675927.1 |
| *Escherichia* phage anhysbys | *Escherichia* *coli* K-12 MG1655 | NC_052656.1 |
| *Escherichia* phage ESCO13 | *Escherichia* *coli* BEN 4311 | NC_047770.1 |
| *Escherichia* phage AnYang | *Escherichia* *coli* O157 | NC_055782.1 |
| *Escherichia* phage teqdroes | *Escherichia* *coli* MG1655 K-12 | NC_054932.1 |
| *Escherichia* phage phT4A | *Escherichia* *coli* ATCC 13706 | NC_055712.1 |
| *Escherichia* phage ukendt | *Escherichia* *coli* K-12 MG1655 | NC_052661.1 |
| *Enterobacteria* phage phi92 | *Escherichia* *coli* K92 | NC_023693.1 |
| *Escherichia* phage vB_vPM_PD06 | *Escherichia* *coli* | NC_052653.1 |
| *Escherichia* phage Rac-SA53 | *Escherichia* *coli* SA53 | KU052037.1 |
| *Escherichia* phage phAPEC8 | *Escherichia* *coli* (virulen avian pathogenic) | NC_020079.1 |
| *Escherichia* phage vB_EcoM_PHB05 | Shiga toxin-producing *Escherichia* *coli* | NC_052652.1 |
