# Supplementary material for: Genomic characterization of bacteriophage BI-EHEC infecting strains of Enterohemorrhagic Escherichia coli
Source: BMC Res Notes. 2021 Dec 20;14:459. doi: 10.1186/s13104-021-05881-5 (PMC8686590; doi:10.1186/s13104-021-05881-5)
Supplement: Supplementary file 4 — Additional file 4. Table S3 US-EHEC CARD results (highest to lowest best identities). [file 13104_2021_5881_MOESM4_ESM.docx]

Table S3 US-EHEC CARD results (highest to lowest best identities)

| Contig | Best Hit ARO | Best Identities | Drug Class | Resistance Mechanism | AMR Gene Family |
| --- | --- | --- | --- | --- | --- |
| contig_1_231 | TriC | 72,73 | triclosan | antibiotic efflux | resistance-nodulation-cell division (RND) antibiotic efflux pump |
| contig_1_8 | tva(A) | 66,67 | macrolide antibiotic; lincosamide antibiotic; streptogramin antibiotic; tetracycline antibiotic; oxazolidinone antibiotic; phenicol antibiotic; pleuromutilin antibiotic | antibiotic target protection | ABC-F ATP-binding cassette ribosomal protection protein |
| contig_1_92 | Mef(En2) | 64,29 | macrolide antibiotic | antibiotic efflux | major facilitator superfamily (MFS) antibiotic efflux pump |
| contig_1_279 | cprS | 63,64 | peptide antibiotic | antibiotic target alteration; antibiotic efflux | pmr phosphoethanolamine transferase |
| contig_1_251 | sul2 | 61,54 | sulfonamide antibiotic | antibiotic target replacement | sulfonamide resistant sul |
| contig_1_257 | OXA-56 | 61,54 | carbapenem; cephalosporin; penam | antibiotic inactivation | OXA beta-lactamase |
| contig_1_266 | aadA4 | 61,54 | aminoglycoside antibiotic | antibiotic inactivation | ANT(3'') |
| contig_1_268 | *Staphylococcus aureus*  mupB conferring resistance to mupirocin | 61,54 | mupirocin | antibiotic target alteration | antibiotic-resistant isoleucyl-tRNA synthetase (ileS) |
| contig_1_189 | mtrD | 58,82 | macrolide antibiotic; penam | antibiotic efflux | resistance-nodulation-cell division (RND) antibiotic efflux pump |
| contig_1_208 | NPS-1 | 56,52 | cephalosporin; penam | antibiotic inactivation | NPS beta-lactamase |
| contig_1_98 | vanWB | 56,25 | glycopeptide antibiotic | antibiotic target alteration | vanW; glycopeptide resistance gene cluster |
| contig_1_120 | OpmD | 55,56 | fluoroquinolone antibiotic; tetracycline antibiotic; acridine dye | antibiotic efflux | resistance-nodulation-cell division (RND) antibiotic efflux pump |
| contig_1_173 | macB | 55 | macrolide antibiotic | antibiotic efflux | ATP-binding cassette (ABC) antibiotic efflux pump |
| contig_1_181 | rmtB | 54,55 | aminoglycoside antibiotic | antibiotic target alteration | 16S rRNA methyltransferase (G1405) |
| contig_1_63 | cmrA | 53,33 | phenicol antibiotic | antibiotic efflux | major facilitator superfamily (MFS) antibiotic efflux pump |
| contig_1_135 | rgt1438 | 52,94 | rifamycin antibiotic | antibiotic inactivation | rifampin glycosyltransferase |
| contig_1_226 | cmlB | 52,94 | phenicol antibiotic | antibiotic efflux | major facilitator superfamily (MFS) antibiotic efflux pump |
| contig_1_271 | OXA-209 | 52,94 | carbapenem; cephalosporin; penam | antibiotic inactivation | OXA beta-lactamase |
| contig_1_91 | adeN | 52,63 | macrolide antibiotic; fluoroquinolone antibiotic; lincosamide antibiotic; carbapenem; cephalosporin; tetracycline antibiotic; rifamycin antibiotic; diaminopyrimidine antibiotic; phenicol antibiotic; penem | antibiotic efflux | resistance-nodulation-cell division (RND) antibiotic efflux pump |
| contig_1_196 | y56 beta-lactamase | 52 | cephalosporin; penam | antibiotic inactivation | BlaA beta-lactamase |
| contig_1_96 | IND-1 | 51,52 | carbapenem | antibiotic inactivation | IND beta-lactamase |
| contig_1_50 | MCR-4.1 | 50 | peptide antibiotic | antibiotic target alteration | MCR phosphoethanolamine transferase |
| contig_1_94 | *Bacillus subtilis* mprF | 50 | peptide antibiotic | antibiotic target alteration | defensin resistant mprF |
| contig_1_124 | mdtP | 50 | nucleoside antibiotic; acridine dye | antibiotic efflux | major facilitator superfamily (MFS) antibiotic efflux pump |
| contig_1_133 | tet(35) | 50 | tetracycline antibiotic | antibiotic efflux | ATP-binding cassette (ABC) antibiotic efflux pump |
| contig_1_194 | evgS | 50 | macrolide antibiotic; fluoroquinolone antibiotic; penam; tetracycline antibiotic | antibiotic efflux | major facilitator superfamily (MFS) antibiotic efflux pump; resistance-nodulation-cell division (RND) antibiotic efflux pump |
| contig_1_222 | vgaC | 50 | macrolide antibiotic; lincosamide antibiotic; streptogramin antibiotic; tetracycline antibiotic; oxazolidinone antibiotic; phenicol antibiotic; pleuromutilin antibiotic | antibiotic target protection | ABC-F ATP-binding cassette ribosomal protection protein |
| contig_1_246 | CMY-103 | 50 | cephamycin | antibiotic inactivation | CMY beta-lactamase |
| contig_1_80 | tetQ | 48,28 | tetracycline antibiotic | antibiotic target protection | tetracycline-resistant ribosomal protection protein |
| contig_1_60 | TMB-2 | 48,15 | carbapenem; cephalosporin; cephamycin | antibiotic inactivation | TMB beta-lactamase |
| contig_1_134 | bcr-1 | 47,83 | bicyclomycin | antibiotic efflux | major facilitator superfamily (MFS) antibiotic efflux pump |
| contig_1_186 | oqxB | 47,83 | fluoroquinolone antibiotic; glycylcycline; tetracycline antibiotic; diaminopyrimidine antibiotic; nitrofuran antibiotic | antibiotic efflux | resistance-nodulation-cell division (RND) antibiotic efflux pump |
| contig_1_28 | SHV-63 | 47,62 | carbapenem; cephalosporin; penam | antibiotic inactivation | SHV beta-lactamase |
| contig_1_256 | Erm(K) | 47,62 | macrolide antibiotic; lincosamide antibiotic; streptogramin antibiotic | antibiotic target alteration | Erm 23S ribosomal RNA methyltransferase |
| contig_1_11 | FIM-1 | 47,37 | carbapenem; cephalosporin | antibiotic inactivation | FIM beta-lactamase |
| contig_1_250 | acrD | 47,37 | aminoglycoside antibiotic | antibiotic efflux | resistance-nodulation-cell division (RND) antibiotic efflux pump |
| contig_1_264 | msrE | 47,37 | macrolide antibiotic; lincosamide antibiotic; streptogramin antibiotic; tetracycline antibiotic; oxazolidinone antibiotic; phenicol antibiotic; pleuromutilin antibiotic | antibiotic target protection | ABC-F ATP-binding cassette ribosomal protection protein |
| contig_1_156 | mecB | 46,34 | penam | antibiotic target replacement | methicillin resistant PBP2 |
| contig_1_74 | tetW | 46,15 | tetracycline antibiotic | antibiotic target protection | tetracycline-resistant ribosomal protection protein |
| contig_1_190 | TriC | 46,15 | triclosan | antibiotic efflux | resistance-nodulation-cell division (RND) antibiotic efflux pump |
| contig_1_277 | LpeA | 46,15 | macrolide antibiotic | antibiotic efflux | resistance-nodulation-cell division (RND) antibiotic efflux pump |
| contig_1_70 | evgS | 45,83 | macrolide antibiotic; fluoroquinolone antibiotic; penam; tetracycline antibiotic | antibiotic efflux | major facilitator superfamily (MFS) antibiotic efflux pump; resistance-nodulation-cell division (RND) antibiotic efflux pump |
| contig_1_241 | tetB(60) | 45,83 | tetracycline antibiotic | antibiotic efflux | ATP-binding cassette (ABC) antibiotic efflux pump |
| contig_1_114 | vgaC | 45,45 | macrolide antibiotic; lincosamide antibiotic; streptogramin antibiotic; tetracycline antibiotic; oxazolidinone antibiotic; phenicol antibiotic; pleuromutilin antibiotic | antibiotic target protection | ABC-F ATP-binding cassette ribosomal protection protein |
| contig_1_2 | MCR-2.2 | 45 | peptide antibiotic | antibiotic target alteration | MCR phosphoethanolamine transferase |
| contig_1_42 | vgaE | 45 | macrolide antibiotic; lincosamide antibiotic; streptogramin antibiotic; tetracycline antibiotic; oxazolidinone antibiotic; phenicol antibiotic; pleuromutilin antibiotic | antibiotic target protection | ABC-F ATP-binding cassette ribosomal protection protein |
| contig_1_275 | vgaALC | 45 | macrolide antibiotic; lincosamide antibiotic; streptogramin antibiotic; tetracycline antibiotic; oxazolidinone antibiotic; phenicol antibiotic; pleuromutilin antibiotic | antibiotic target protection | ABC-F ATP-binding cassette ribosomal protection protein |
| contig_1_47 | IDC-1 | 44,83 | carbapenem; cephalosporin | antibiotic inactivation | IDC beta-lactamase |
| contig_1_6 | APH(3')-Vc | 44,44 | aminoglycoside antibiotic | antibiotic inactivation | APH(3') |
| contig_1_48 | cmlB | 44,44 | phenicol antibiotic | antibiotic efflux | major facilitator superfamily (MFS) antibiotic efflux pump |
| contig_1_203 | cmeA | 44,44 | macrolide antibiotic; fluoroquinolone antibiotic; cephalosporin; fusidic acid | antibiotic efflux | resistance-nodulation-cell division (RND) antibiotic efflux pump |
| contig_1_37 | msrE | 44,12 | macrolide antibiotic; lincosamide antibiotic; streptogramin antibiotic; tetracycline antibiotic; oxazolidinone antibiotic; phenicol antibiotic; pleuromutilin antibiotic | antibiotic target protection | ABC-F ATP-binding cassette ribosomal protection protein |
| contig_1_69 | LpeB | 44 | macrolide antibiotic | antibiotic efflux | resistance-nodulation-cell division (RND) antibiotic efflux pump |
| contig_1_100 | catB8 | 44 | phenicol antibiotic | antibiotic inactivation | chloramphenicol acetyltransferase (CAT) |
| contig_1_187 | *Enterococcus faecalis* chloramphenicol acetyltransferase | 44 | phenicol antibiotic | antibiotic inactivation | chloramphenicol acetyltransferase (CAT) |
| contig_1_160 | mdtF | 43,75 | macrolide antibiotic; fluoroquinolone antibiotic; penam | antibiotic efflux | resistance-nodulation-cell division (RND) antibiotic efflux pump |
| contig_1_112 | SHV-173 | 43,48 | carbapenem; cephalosporin; penam | antibiotic inactivation | SHV beta-lactamase |
| contig_1_227 | MexV | 43,48 | macrolide antibiotic; fluoroquinolone antibiotic; tetracycline antibiotic; acridine dye; phenicol antibiotic | antibiotic efflux | resistance-nodulation-cell division (RND) antibiotic efflux pump |
| contig_1_229 | *Bifidobacterium adolescentis* rpoB mutants conferring resistance to rifampicin | 43,48 | rifamycin antibiotic | antibiotic target alteration; antibiotic target replacement | rifamycin-resistant beta-subunit of RNA polymerase (rpoB) |
| contig_1_30 | *Staphylococcus aureus*  mupA conferring resistance to mupirocin | 42,86 | mupirocin | antibiotic target alteration | antibiotic-resistant isoleucyl-tRNA synthetase (ileS) |
| contig_1_125 | RCP-1 | 42,86 | penam | antibiotic inactivation | RCP beta-lactamase |
| contig_1_153 | LpsA | 42,86 | peptide antibiotic | reduced permeability to antibiotic | Intrinsic peptide antibiotic resistant Lps |
| contig_1_212 | *Vibrio cholerae* varG | 42,86 | carbapenem | antibiotic inactivation | subclass B1 *Vibrio cholerae* varG beta-lactamase |
| contig_1_76 | SHV-16 | 42,11 | carbapenem; cephalosporin; penam | antibiotic inactivation | SHV beta-lactamase |
| contig_1_260 | msrH | 42,11 | macrolide antibiotic; lincosamide antibiotic; streptogramin antibiotic; tetracycline antibiotic; oxazolidinone antibiotic; phenicol antibiotic; pleuromutilin antibiotic | antibiotic target protection | ABC-F ATP-binding cassette ribosomal protection protein |
| contig_1_247 | salA | 41,94 | macrolide antibiotic; lincosamide antibiotic; streptogramin antibiotic; tetracycline antibiotic; oxazolidinone antibiotic; phenicol antibiotic; pleuromutilin antibiotic | antibiotic target protection | ABC-F ATP-binding cassette ribosomal protection protein |
| contig_1_16 | msrC | 41,67 | macrolide antibiotic; lincosamide antibiotic; streptogramin antibiotic; tetracycline antibiotic; oxazolidinone antibiotic; phenicol antibiotic; pleuromutilin antibiotic | antibiotic target protection | ABC-F ATP-binding cassette ribosomal protection protein |
| contig_1_104 | patA | 41,67 | fluoroquinolone antibiotic | antibiotic efflux | ATP-binding cassette (ABC) antibiotic efflux pump |
| contig_1_233 | chrB | 41,67 | macrolide antibiotic; lincosamide antibiotic | antibiotic target alteration | non-erm 23S ribosomal RNA methyltransferase (G748) |
| contig_1_244 | *Bifidobacterium bifidum* ileS conferring resistance to mupirocin | 41,38 | mupirocin | antibiotic target alteration | antibiotic-resistant isoleucyl-tRNA synthetase (ileS) |
| contig_1_255 | vanF | 41,38 | glycopeptide antibiotic | antibiotic target alteration | glycopeptide resistance gene cluster; van ligase |
| contig_1_201 | APH(6)-Id | 41,03 | aminoglycoside antibiotic | antibiotic inactivation | APH(6) |
| contig_1_17 | vanRF | 40,91 | glycopeptide antibiotic | antibiotic target alteration | glycopeptide resistance gene cluster; vanR |
| contig_1_143 | FosC | 40,91 | fosfomycin | antibiotic inactivation | fosC phosphotransferase family |
| contig_1_118 | IND-10 | 40,74 | carbapenem | antibiotic inactivation | IND beta-lactamase |
| contig_1_202 | ADC-42 | 40,54 | cephalosporin | antibiotic inactivation | ADC beta-lactamase without carbapenemase activity |
| contig_1_131 | cfrA | 40,48 | lincosamide antibiotic; streptogramin antibiotic; oxazolidinone antibiotic; phenicol antibiotic; pleuromutilin antibiotic | antibiotic target alteration | Cfr 23S ribosomal RNA methyltransferase |
| contig_1_41 | cmlA4 | 40 | phenicol antibiotic | antibiotic efflux | major facilitator superfamily (MFS) antibiotic efflux pump |
| contig_1_67 | cmeB | 40 | macrolide antibiotic; fluoroquinolone antibiotic; cephalosporin; fusidic acid | antibiotic efflux | resistance-nodulation-cell division (RND) antibiotic efflux pump |
| contig_1_180 | msrH | 40 | macrolide antibiotic; lincosamide antibiotic; streptogramin antibiotic; tetracycline antibiotic; oxazolidinone antibiotic; phenicol antibiotic; pleuromutilin antibiotic | antibiotic target protection | ABC-F ATP-binding cassette ribosomal protection protein |
| contig_1_54 | OXA-12 | 39,39 | carbapenem; cephalosporin; penam | antibiotic inactivation | OXA beta-lactamase |
| contig_1_197 | tetW | 39,39 | tetracycline antibiotic | antibiotic target protection | tetracycline-resistant ribosomal protection protein |
| contig_1_127 | tetA(46) | 39,29 | tetracycline antibiotic | antibiotic efflux | ATP-binding cassette (ABC) antibiotic efflux pump |
| contig_1_168 | *Chlamydia trachomatis* intrinsic murA conferring resistance to fosfomycin | 39,13 | fosfomycin | antibiotic target alteration | antibiotic-resistant murA transferase |
| contig_1_121 | CMY-27 | 38,71 | cephamycin | antibiotic inactivation | CMY beta-lactamase |
| contig_1_46 | CAM-1 | 38,24 | carbapenem; cephalosporin; cephamycin; penam | antibiotic inactivation | CAM beta-lactamase |
| contig_1_56 | TaeA | 38,24 | pleuromutilin antibiotic | antibiotic efflux | ATP-binding cassette (ABC) antibiotic efflux pump |
| contig_1_258 | floR | 38,1 | phenicol antibiotic | antibiotic efflux | major facilitator superfamily (MFS) antibiotic efflux pump |
| contig_1_270 | adeS | 38,1 | glycylcycline; tetracycline antibiotic | antibiotic efflux | resistance-nodulation-cell division (RND) antibiotic efflux pump |
| contig_1_7 | rphB | 37,93 | rifamycin antibiotic | antibiotic inactivation | rifampin phosphotransferase |
| contig_1_228 | MexF | 37,93 | fluoroquinolone antibiotic; diaminopyrimidine antibiotic; phenicol antibiotic | antibiotic efflux | resistance-nodulation-cell division (RND) antibiotic efflux pump |
| contig_1_191 | amrB | 37,84 | aminoglycoside antibiotic | antibiotic efflux | resistance-nodulation-cell division (RND) antibiotic efflux pump |
| contig_1_52 | cipA | 37,78 | lincosamide antibiotic; streptogramin antibiotic; oxazolidinone antibiotic; phenicol antibiotic; pleuromutilin antibiotic | antibiotic target alteration | Cfr 23S ribosomal RNA methyltransferase |
| contig_1_97 | MexF | 37,78 | fluoroquinolone antibiotic; diaminopyrimidine antibiotic; phenicol antibiotic | antibiotic efflux | resistance-nodulation-cell division (RND) antibiotic efflux pump |
| contig_1_218 | lsaA | 37,78 | macrolide antibiotic; lincosamide antibiotic; streptogramin antibiotic; tetracycline antibiotic; oxazolidinone antibiotic; phenicol antibiotic; pleuromutilin antibiotic | antibiotic target protection | ABC-F ATP-binding cassette ribosomal protection protein |
| contig_1_22 | AAC(6')-30/AAC(6')-Ib' fusion protein | 37,5 | aminoglycoside antibiotic | antibiotic inactivation | AAC(6') |
| contig_1_115 | MexW | 37,5 | macrolide antibiotic; fluoroquinolone antibiotic; tetracycline antibiotic; acridine dye; phenicol antibiotic | antibiotic efflux | resistance-nodulation-cell division (RND) antibiotic efflux pump |
| contig_1_123 | MCR-4.3 | 37,5 | peptide antibiotic | antibiotic target alteration | MCR phosphoethanolamine transferase |
| contig_1_165 | OXA-303 | 37,5 | carbapenem; cephalosporin; penam | antibiotic inactivation | OXA beta-lactamase |
| contig_1_265 | APH(2'')-If | 37,14 | aminoglycoside antibiotic | antibiotic inactivation | APH(2'') |
| contig_1_20 | TaeA | 37,04 | pleuromutilin antibiotic | antibiotic efflux | ATP-binding cassette (ABC) antibiotic efflux pump |
| contig_1_184 | AAC(6')-Ian | 36,84 | aminoglycoside antibiotic | antibiotic inactivation | AAC(6') |
| contig_1_205 | basS | 36,73 | peptide antibiotic | antibiotic target alteration; antibiotic efflux | pmr phosphoethanolamine transferase |
| contig_1_138 | rosB | 36,67 | peptide antibiotic | antibiotic efflux | major facilitator superfamily (MFS) antibiotic efflux pump |
| contig_1_157 | macB | 36,59 | macrolide antibiotic | antibiotic efflux | ATP-binding cassette (ABC) antibiotic efflux pump |
| contig_1_198 | DHA-12 | 36,36 | cephalosporin; cephamycin | antibiotic inactivation | DHA beta-lactamase |
| contig_1_65 | LRA-3 | 36 | cephalosporin; penam | antibiotic inactivation | subclass B3 LRA beta-lactamase |
| contig_1_113 | LpsB | 35,9 | peptide antibiotic | reduced permeability to antibiotic | Intrinsic peptide antibiotic resistant Lps |
| contig_1_136 | AAC(6')-31 | 35,71 | aminoglycoside antibiotic | antibiotic inactivation | AAC(6') |
| contig_1_259 | vanSO | 35,71 | glycopeptide antibiotic | antibiotic target alteration | vanS; glycopeptide resistance gene cluster |
| contig_1_282 | CfxA5 | 35,71 | cephamycin | antibiotic inactivation | CfxA beta-lactamase |
| contig_1_14 | tet(55) | 35,56 | tetracycline antibiotic | antibiotic inactivation | tetracycline inactivation enzyme |
| contig_1_111 | mdtC | 35,48 | aminocoumarin antibiotic | antibiotic efflux | resistance-nodulation-cell division (RND) antibiotic efflux pump |
| contig_1_144 | vanRN | 35,48 | glycopeptide antibiotic | antibiotic target alteration | glycopeptide resistance gene cluster; vanR |
| contig_1_223 | smeD | 35,48 | macrolide antibiotic; fluoroquinolone antibiotic; tetracycline antibiotic; phenicol antibiotic | antibiotic efflux | resistance-nodulation-cell division (RND) antibiotic efflux pump |
| contig_1_109 | emrY | 35,42 | tetracycline antibiotic | antibiotic efflux | major facilitator superfamily (MFS) antibiotic efflux pump |
| contig_1_31 | ICR-Mo | 35,29 | peptide antibiotic | antibiotic target alteration | intrinsic colistin resistant phosphoethanolamine transferase |
| contig_1_49 | arlS | 35,14 | fluoroquinolone antibiotic; acridine dye | antibiotic efflux | major facilitator superfamily (MFS) antibiotic efflux pump |
| contig_1_253 | dfrA20 | 35,14 | diaminopyrimidine antibiotic | antibiotic target replacement | trimethoprim resistant dihydrofolate reductase dfr |
| contig_1_204 | lmrD | 34,78 | lincosamide antibiotic | antibiotic efflux | ATP-binding cassette (ABC) antibiotic efflux pump |
| contig_1_213 | AQU-1 | 34,78 | cephalosporin | antibiotic inactivation | AQU beta-lactamase |
| contig_1_207 | MexD | 34,62 | macrolide antibiotic; fluoroquinolone antibiotic; aminoglycoside antibiotic; cephalosporin; penam; tetracycline antibiotic; aminocoumarin antibiotic; diaminopyrimidine antibiotic; phenicol antibiotic | antibiotic efflux | resistance-nodulation-cell division (RND) antibiotic efflux pump |
| contig_1_276 | mdsB | 34,62 | monobactam; carbapenem; cephalosporin; cephamycin; penam; phenicol antibiotic; penem | antibiotic efflux | resistance-nodulation-cell division (RND) antibiotic efflux pump |
| contig_1_163 | clbC | 34,55 | lincosamide antibiotic; streptogramin antibiotic; oxazolidinone antibiotic; phenicol antibiotic; pleuromutilin antibiotic | antibiotic target alteration | Cfr 23S ribosomal RNA methyltransferase |
| contig_1_200 | MexE | 34,48 | fluoroquinolone antibiotic; diaminopyrimidine antibiotic; phenicol antibiotic | antibiotic efflux | resistance-nodulation-cell division (RND) antibiotic efflux pump |
| contig_1_216 | ykkD | 34,48 | aminoglycoside antibiotic; tetracycline antibiotic; phenicol antibiotic | antibiotic efflux | small multidrug resistance (SMR) antibiotic efflux pump |
| contig_1_107 | OXA-465 | 34,38 | carbapenem; cephalosporin; penam | antibiotic inactivation | OXA beta-lactamase |
| contig_1_29 | vgaE | 34,29 | macrolide antibiotic; lincosamide antibiotic; streptogramin antibiotic; tetracycline antibiotic; oxazolidinone antibiotic; phenicol antibiotic; pleuromutilin antibiotic | antibiotic target protection | ABC-F ATP-binding cassette ribosomal protection protein |
| contig_1_235 | otr(B) | 34,29 | tetracycline antibiotic | antibiotic efflux | major facilitator superfamily (MFS) antibiotic efflux pump |
| contig_1_159 | OXA-476 | 34,18 | carbapenem; cephalosporin; penam | antibiotic inactivation | OXA beta-lactamase |
| contig_1_35 | LpeB | 33,9 | macrolide antibiotic | antibiotic efflux | resistance-nodulation-cell division (RND) antibiotic efflux pump |
| contig_1_19 | vanJ | 33,33 | glycopeptide antibiotic | antibiotic target alteration | vanJ membrane protein |
| contig_1_39 | cmeA | 33,33 | macrolide antibiotic; fluoroquinolone antibiotic; cephalosporin; fusidic acid | antibiotic efflux | resistance-nodulation-cell division (RND) antibiotic efflux pump |
| contig_1_40 | *Bifidobacterium adolescentis* rpoB mutants conferring resistance to rifampicin | 33,33 | rifamycin antibiotic | antibiotic target alteration; antibiotic target replacement | rifamycin-resistant beta-subunit of RNA polymerase (rpoB) |
| contig_1_84 | AAC(6')-33 | 33,33 | aminoglycoside antibiotic | antibiotic inactivation | AAC(6') |
| contig_1_89 | FosX | 33,33 | fosfomycin | antibiotic inactivation | fosfomycin thiol transferase |
| contig_1_102 | abcA | 33,33 | cephalosporin; penam; peptide antibiotic | antibiotic efflux | ATP-binding cassette (ABC) antibiotic efflux pump |
| contig_1_122 | LptD | 33,33 | carbapenem; peptide antibiotic; aminocoumarin antibiotic; rifamycin antibiotic | antibiotic efflux | ATP-binding cassette (ABC) antibiotic efflux pump |
| contig_1_139 | OXA-129 | 33,33 | carbapenem; cephalosporin; penam | antibiotic inactivation | OXA beta-lactamase |
| contig_1_167 | CAU-1 | 33,33 | carbapenem; penam | antibiotic inactivation | CAU beta-lactamase |
| contig_1_232 | dfrD | 33,33 | diaminopyrimidine antibiotic | antibiotic target replacement | trimethoprim resistant dihydrofolate reductase dfr |
| contig_1_21 | FosA3 | 32,81 | fosfomycin | antibiotic inactivation | fosfomycin thiol transferase |
| contig_1_137 | Erm(47) | 32,73 | macrolide antibiotic; lincosamide antibiotic; streptogramin antibiotic | antibiotic target alteration | Erm 23S ribosomal RNA methyltransferase |
| contig_1_68 | rphB | 32,69 | rifamycin antibiotic | antibiotic inactivation | rifampin phosphotransferase |
| contig_1_79 | *Pseudomonas aeruginosa* catB7 | 32,69 | phenicol antibiotic | antibiotic inactivation | chloramphenicol acetyltransferase (CAT) |
| contig_1_117 | ANT(3'')-IIb | 32,69 | aminoglycoside antibiotic | antibiotic inactivation | ANT(3'') |
| contig_1_274 | PEDO-3 | 32,56 | carbapenem | antibiotic inactivation | subclass B1 PEDO beta-lactamase |
| contig_1_55 | Yrc-1 | 32,5 | cephalosporin; penam | antibiotic inactivation | YRC Beta-lactamase |
| contig_1_269 | mecC-type BlaZ | 32,5 | penam | antibiotic inactivation | blaZ beta-lactamase |
| contig_1_280 | TEM-146 | 32,5 | monobactam; cephalosporin; penam; penem | antibiotic inactivation | TEM beta-lactamase |
| contig_1_132 | ROB-6 | 32,26 | cephalosporin; penam | antibiotic inactivation | ROB beta-lactamase |
| contig_1_237 | vanHM | 32,2 | glycopeptide antibiotic | antibiotic target alteration | vanH; glycopeptide resistance gene cluster |
| contig_1_169 | *Clostridium butyricum* catB | 32,14 | phenicol antibiotic | antibiotic inactivation | chloramphenicol acetyltransferase (CAT) |
| contig_1_25 | JOHN-1 | 32,08 | carbapenem; cephalosporin; penam | antibiotic inactivation | JOHN beta-lactamase |
| contig_1_174 | tva(A) | 32 | macrolide antibiotic; lincosamide antibiotic; streptogramin antibiotic; tetracycline antibiotic; oxazolidinone antibiotic; phenicol antibiotic; pleuromutilin antibiotic | antibiotic target protection | ABC-F ATP-binding cassette ribosomal protection protein |
| contig_1_175 | OXA-326 | 32 | carbapenem; cephalosporin; penam | antibiotic inactivation | OXA beta-lactamase |
| contig_1_278 | AcrS | 32 | fluoroquinolone antibiotic; cephalosporin; glycylcycline; cephamycin; penam; tetracycline antibiotic; rifamycin antibiotic; phenicol antibiotic; triclosan | antibiotic efflux | resistance-nodulation-cell division (RND) antibiotic efflux pump |
| contig_1_158 | macB | 31,91 | macrolide antibiotic | antibiotic efflux | ATP-binding cassette (ABC) antibiotic efflux pump |
| contig_1_62 | cphA4 | 31,71 | carbapenem | antibiotic inactivation | CphA beta-lactamase |
| contig_1_182 | mecC-type BlaZ | 31,71 | penam | antibiotic inactivation | blaZ beta-lactamase |
| contig_1_171 | mtrE | 31,67 | macrolide antibiotic; penam | antibiotic efflux | resistance-nodulation-cell division (RND) antibiotic efflux pump |
| contig_1_26 | otr(A) | 31,58 | tetracycline antibiotic | antibiotic target protection | tetracycline-resistant ribosomal protection protein |
| contig_1_273 | EreB | 31,43 | macrolide antibiotic | antibiotic inactivation | macrolide esterase |
| contig_1_93 | macB | 31,25 | macrolide antibiotic | antibiotic efflux | ATP-binding cassette (ABC) antibiotic efflux pump |
| contig_1_162 | ceoB | 31,25 | fluoroquinolone antibiotic; aminoglycoside antibiotic | antibiotic efflux | resistance-nodulation-cell division (RND) antibiotic efflux pump |
| contig_1_103 | EdeQ | 31,15 | peptide antibiotic; polyamine antibiotic | antibiotic inactivation | Edeine acetyltransferase |
| contig_1_148 | OXA-475 | 31,11 | carbapenem; cephalosporin; penam | antibiotic inactivation | OXA beta-lactamase |
| contig_1_172 | mtrD | 31,11 | macrolide antibiotic; penam | antibiotic efflux | resistance-nodulation-cell division (RND) antibiotic efflux pump |
| contig_1_166 | THIN-B | 31,03 | carbapenem; cephalosporin; penam | antibiotic inactivation | THIN-B beta-lactamase |
| contig_1_66 | AAC(6')-Iad | 30,95 | aminoglycoside antibiotic | antibiotic inactivation | AAC(6') |
| contig_1_44 | AcrF | 30,91 | fluoroquinolone antibiotic; cephalosporin; cephamycin; penam | antibiotic efflux | resistance-nodulation-cell division (RND) antibiotic efflux pump |
| contig_1_36 | AAC(3)-Ia | 30,77 | aminoglycoside antibiotic | antibiotic inactivation | AAC(3) |
| contig_1_108 | tap | 30,77 | tetracycline antibiotic | antibiotic efflux | major facilitator superfamily (MFS) antibiotic efflux pump |
| contig_1_147 | IMP-52 | 30,77 | carbapenem; cephalosporin; cephamycin; penam; penem | antibiotic inactivation | IMP beta-lactamase |
| contig_1_161 | vanHD | 30,38 | glycopeptide antibiotic | antibiotic target alteration | vanH; glycopeptide resistance gene cluster |
| contig_1_151 | tetA(60) | 30,3 | tetracycline antibiotic | antibiotic efflux | ATP-binding cassette (ABC) antibiotic efflux pump |
| contig_1_243 | OmpA | 30,23 | monobactam; carbapenem; cephalosporin; cephamycin; penam; penem | reduced permeability to antibiotic | General Bacterial Porin with reduced permeability to beta-lactams |
| contig_1_155 | gimA | 30 | macrolide antibiotic | antibiotic inactivation | gimA family macrolide glycosyltransferase |
| contig_1_195 | rpoB2 | 30 | rifamycin antibiotic | antibiotic target alteration; antibiotic target replacement | rifamycin-resistant beta-subunit of RNA polymerase (rpoB) |
| contig_1_239 | CTX-M-41 | 30 | cephalosporin | antibiotic inactivation | CTX-M beta-lactamase |
| contig_1_221 | otrC | 29,79 | tetracycline antibiotic | antibiotic efflux | ATP-binding cassette (ABC) antibiotic efflux pump |
| contig_1_149 | AAC(6')-Iad | 29,63 | aminoglycoside antibiotic | antibiotic inactivation | AAC(6') |
| contig_1_24 | CMY-76 | 29,51 | cephamycin | antibiotic inactivation | CMY beta-lactamase |
| contig_1_12 | ceoB | 29,41 | fluoroquinolone antibiotic; aminoglycoside antibiotic | antibiotic efflux | resistance-nodulation-cell division (RND) antibiotic efflux pump |
| contig_1_283 | vanXB | 29,31 | glycopeptide antibiotic | antibiotic target alteration | vanX; glycopeptide resistance gene cluster |
| contig_1_4 | MCR-3.2 | 29,17 | peptide antibiotic | antibiotic target alteration | MCR phosphoethanolamine transferase |
| contig_1_85 | arlS | 29,17 | fluoroquinolone antibiotic; acridine dye | antibiotic efflux | major facilitator superfamily (MFS) antibiotic efflux pump |
| contig_1_23 | vanTG | 29,03 | glycopeptide antibiotic | antibiotic target alteration | glycopeptide resistance gene cluster; vanT |
| contig_1_267 | AAC(6')-32 | 29,03 | aminoglycoside antibiotic | antibiotic inactivation | AAC(6') |
| contig_1_152 | ARL-6 | 28,89 | penam | antibiotic inactivation | ARL Beta-lactamase |
| contig_1_209 | PEDO-1 | 28,89 | carbapenem | antibiotic inactivation | subclass B3 PEDO beta-lactamase |
| contig_1_99 | BahA | 28,81 | peptide antibiotic | antibiotic inactivation | Bah amidohydrolase |
| contig_1_9 | ermZ | 28,57 | macrolide antibiotic; lincosamide antibiotic; streptogramin antibiotic | antibiotic target alteration | Erm 23S ribosomal RNA methyltransferase |
| contig_1_18 | MuxC | 28,57 | macrolide antibiotic; monobactam; tetracycline antibiotic; aminocoumarin antibiotic | antibiotic efflux | resistance-nodulation-cell division (RND) antibiotic efflux pump |
| contig_1_27 | dfrA13 | 28,57 | diaminopyrimidine antibiotic | antibiotic target replacement | trimethoprim resistant dihydrofolate reductase dfr |
| contig_1_51 | oleD | 28,57 | macrolide antibiotic | antibiotic inactivation | ole glycosyltransferase |
| contig_1_119 | *Bifidobacterium adolescentis* rpoB mutants conferring resistance to rifampicin | 28,57 | rifamycin antibiotic | antibiotic target alteration; antibiotic target replacement | rifamycin-resistant beta-subunit of RNA polymerase (rpoB) |
| contig_1_150 | MexK | 28,57 | macrolide antibiotic; tetracycline antibiotic; triclosan | antibiotic efflux | resistance-nodulation-cell division (RND) antibiotic efflux pump |
| contig_1_106 | MIR-4 | 28,38 | monobactam; cephalosporin | antibiotic inactivation | MIR beta-lactamase |
| contig_1_75 | ceoB | 28,3 | fluoroquinolone antibiotic; aminoglycoside antibiotic | antibiotic efflux | resistance-nodulation-cell division (RND) antibiotic efflux pump |
| contig_1_206 | vanO | 28,21 | glycopeptide antibiotic | antibiotic target alteration | glycopeptide resistance gene cluster; van ligase |
| contig_1_281 | mel | 28,12 | macrolide antibiotic; lincosamide antibiotic; streptogramin antibiotic; tetracycline antibiotic; oxazolidinone antibiotic; phenicol antibiotic; pleuromutilin antibiotic | antibiotic target protection | ABC-F ATP-binding cassette ribosomal protection protein |
| contig_1_90 | dfrA1 | 28 | diaminopyrimidine antibiotic | antibiotic target replacement | trimethoprim resistant dihydrofolate reductase dfr |
| contig_1_129 | OXA-53 | 27,94 | carbapenem; cephalosporin; penam | antibiotic inactivation | OXA beta-lactamase |
| contig_1_179 | mecR1 | 27,91 | penam | antibiotic target replacement | methicillin resistant PBP2 |
| contig_1_177 | OXA-18 | 27,66 | carbapenem; cephalosporin; penam | antibiotic inactivation | OXA beta-lactamase |
| contig_1_72 | vanSG | 27,59 | glycopeptide antibiotic | antibiotic target alteration | vanS; glycopeptide resistance gene cluster |
| contig_1_126 | EreD | 27,59 | macrolide antibiotic | antibiotic inactivation | macrolide esterase |
| contig_1_234 | lsaA | 27,55 | macrolide antibiotic; lincosamide antibiotic; streptogramin antibiotic; tetracycline antibiotic; oxazolidinone antibiotic; phenicol antibiotic; pleuromutilin antibiotic | antibiotic target protection | ABC-F ATP-binding cassette ribosomal protection protein |
| contig_1_242 | acrD | 27,45 | aminoglycoside antibiotic | antibiotic efflux | resistance-nodulation-cell division (RND) antibiotic efflux pump |
| contig_1_220 | vanA | 27,4 | glycopeptide antibiotic | antibiotic target alteration | glycopeptide resistance gene cluster; van ligase |
| contig_1_10 | lsa(D) | 27,27 | macrolide antibiotic; lincosamide antibiotic; streptogramin antibiotic; tetracycline antibiotic; oxazolidinone antibiotic; phenicol antibiotic; pleuromutilin antibiotic | antibiotic target protection | ABC-F ATP-binding cassette ribosomal protection protein |
| contig_1_81 | mdtN | 27,27 | nucleoside antibiotic; acridine dye | antibiotic efflux | major facilitator superfamily (MFS) antibiotic efflux pump |
| contig_1_261 | OXA-209 | 27,27 | carbapenem; cephalosporin; penam | antibiotic inactivation | OXA beta-lactamase |
| contig_1_83 | CTX-M-152 | 27,12 | cephalosporin | antibiotic inactivation | CTX-M beta-lactamase |
| contig_1_215 | vanI | 27,12 | glycopeptide antibiotic | antibiotic target alteration | glycopeptide resistance gene cluster; van ligase |
| contig_1_73 | LRA-5 | 27,1 | cephalosporin; penam | antibiotic inactivation | class A LRA beta-lactamase |
| contig_1_130 | ICR-Mc | 27,03 | peptide antibiotic | antibiotic target alteration | intrinsic colistin resistant phosphoethanolamine transferase |
| contig_1_59 | vanE | 26,98 | glycopeptide antibiotic | antibiotic target alteration | glycopeptide resistance gene cluster; van ligase |
| contig_1_87 | GES-18 | 26,92 | carbapenem; cephalosporin; penam | antibiotic inactivation | GES beta-lactamase |
| contig_1_43 | *Acinetobacter baumannii* AbaQ | 26,87 | fluoroquinolone antibiotic | antibiotic efflux | major facilitator superfamily (MFS) antibiotic efflux pump |
| contig_1_140 | IMP-48 | 26,85 | carbapenem; cephalosporin; cephamycin; penam; penem | antibiotic inactivation | IMP beta-lactamase |
| contig_1_57 | smeE | 26,79 | macrolide antibiotic; fluoroquinolone antibiotic; tetracycline antibiotic; phenicol antibiotic | antibiotic efflux | resistance-nodulation-cell division (RND) antibiotic efflux pump |
| contig_1_170 | vanYM | 26,67 | glycopeptide antibiotic | antibiotic target alteration | vanY; glycopeptide resistance gene cluster |
| contig_1_263 | LlmA 23S ribosomal RNA methyltransferase | 26,67 | lincosamide antibiotic | antibiotic target alteration | Llm 23S ribosomal RNA methyltransferase |
| contig_1_3 | MexI | 26,56 | fluoroquinolone antibiotic; tetracycline antibiotic; acridine dye | antibiotic efflux | resistance-nodulation-cell division (RND) antibiotic efflux pump |
| contig_1_77 | *Escherichia coli* acrR with mutation conferring multidrug antibiotic resistance | 26,32 | fluoroquinolone antibiotic; cephalosporin; glycylcycline; penam; tetracycline antibiotic; rifamycin antibiotic; phenicol antibiotic; triclosan | antibiotic target alteration; antibiotic efflux | resistance-nodulation-cell division (RND) antibiotic efflux pump |
| contig_1_193 | lmrP | 26,32 | macrolide antibiotic; lincosamide antibiotic; streptogramin antibiotic; tetracycline antibiotic | antibiotic efflux | major facilitator superfamily (MFS) antibiotic efflux pump |
| contig_1_128 | norA | 26,19 | fluoroquinolone antibiotic; acridine dye | antibiotic efflux | major facilitator superfamily (MFS) antibiotic efflux pump |
| contig_1_236 | OXA-9 | 26,05 | carbapenem; cephalosporin; penam | antibiotic inactivation | OXA beta-lactamase |
| contig_1_178 | ErmE | 25,97 | macrolide antibiotic; lincosamide antibiotic; streptogramin antibiotic | antibiotic target alteration | Erm 23S ribosomal RNA methyltransferase |
| contig_1_249 | vanHB | 25,97 | glycopeptide antibiotic | antibiotic target alteration | vanH; glycopeptide resistance gene cluster |
| contig_1_61 | lsa(D) | 25,93 | macrolide antibiotic; lincosamide antibiotic; streptogramin antibiotic; tetracycline antibiotic; oxazolidinone antibiotic; phenicol antibiotic; pleuromutilin antibiotic | antibiotic target protection | ABC-F ATP-binding cassette ribosomal protection protein |
| contig_1_32 | SLB-1 | 25,81 | cephalosporin; penam | antibiotic inactivation | SHW beta-lactamase |
| contig_1_214 | OXA-9 | 25,71 | carbapenem; cephalosporin; penam | antibiotic inactivation | OXA beta-lactamase |
| contig_1_224 | CARB-23 | 25,64 | penam | antibiotic inactivation | CARB beta-lactamase |
| contig_1_252 | RCP-1 | 25,37 | penam | antibiotic inactivation | RCP beta-lactamase |
| contig_1_13 | tet(56) | 25 | tetracycline antibiotic | antibiotic inactivation | tetracycline inactivation enzyme |
| contig_1_86 | MexI | 25 | fluoroquinolone antibiotic; tetracycline antibiotic; acridine dye | antibiotic efflux | resistance-nodulation-cell division (RND) antibiotic efflux pump |
| contig_1_142 | AcrF | 25 | fluoroquinolone antibiotic; cephalosporin; cephamycin; penam | antibiotic efflux | resistance-nodulation-cell division (RND) antibiotic efflux pump |
| contig_1_188 | OXA-85 | 25 | carbapenem; cephalosporin; penam | antibiotic inactivation | OXA beta-lactamase |
| contig_1_58 | adeS | 24,77 | glycylcycline; tetracycline antibiotic | antibiotic efflux | resistance-nodulation-cell division (RND) antibiotic efflux pump |
| contig_1_34 | JOHN-1 | 24,68 | carbapenem; cephalosporin; penam | antibiotic inactivation | JOHN beta-lactamase |
| contig_1_71 | *Staphylococcus aureus*  mupB conferring resistance to mupirocin | 24,32 | mupirocin | antibiotic target alteration | antibiotic-resistant isoleucyl-tRNA synthetase (ileS) |
| contig_1_245 | emrY | 23,81 | tetracycline antibiotic | antibiotic efflux | major facilitator superfamily (MFS) antibiotic efflux pump |
| contig_1_211 | OCH-3 | 22,68 | monobactam; cephalosporin; cephamycin; penam; penem | antibiotic inactivation | OCH beta-lactamase |
| contig_1_5 | OXA-278 | 22,62 | carbapenem; cephalosporin; penam | antibiotic inactivation | OXA beta-lactamase |
| contig_1_101 | MCR-8.1 | 22,5 | peptide antibiotic | antibiotic target alteration | MCR phosphoethanolamine transferase |
| contig_1_164 | lin | 22,45 | lincosamide antibiotic | antibiotic inactivation | lincosamide nucleotidyltransferase (LNU) |
| contig_1_210 | OXA-397 | 22,22 | carbapenem; cephalosporin; penam | antibiotic inactivation | OXA beta-lactamase |
| contig_1_105 | tet(56) | 20,11 | tetracycline antibiotic | antibiotic inactivation | tetracycline inactivation enzyme |
| contig_1_145 | ErmO-srmA | 18,09 | macrolide antibiotic; lincosamide antibiotic; streptogramin antibiotic | antibiotic target alteration | Erm 23S ribosomal RNA methyltransferase |
| contig_1_116 | OXA-447 | 17,36 | carbapenem; cephalosporin; penam | antibiotic inactivation | OXA beta-lactamase |
